# *Akkermansia muciniphila* enhances chicken resistance to infectious bronchitis virus by boosting GABA synthesis and suppressing the NF‐κB inflammatory pathway

**DOI:** 10.1002/imo2.70086

**Published:** 2026-02-23

**Authors:** Ouyang Peng, Yufang Liu, Xuanci Wang, Yihui Huang, Yiqin Yang, Qixiang Kang, Rui Geng, Guangli Hu, Aiping Lv, Fangyu Hu, Yongbo Xia, Xin Luo, Jiamin Liao, Yihong He, Zhanpeng Kuang, Yunping Du, Yun Zhang, Chunyi Xue, Zongxi Han, Wen‐Jun Li, Lei Dong, Hao Zhang, Yongchang Cao

**Affiliations:** ^1^ State Key Laboratory of Biocontrol, School of Life Sciences Sun Yat‐sen University Guangzhou China; ^2^ Guangdong Enterprise Key Laboratory for Animal Health and Environmental Control Wens Foodstuff Group Co. Ltd Yunfu China; ^3^ Hainan University and Massey University International College Haikou China; ^4^ Division of Avian Infectious Diseases, the State Key Laboratory of Animal Disease Prevention and Control, Harbin Veterinary Research Institute the Chinese Academy of Agricultural Sciences Harbin China

**Keywords:** *Akkermansia muciniphila*, antibiotic‐free farming, antiviral mechanistic investigation, fecal microbiota transplantation, gut microbiota, infectious bronchitis virus

## Abstract

Infectious bronchitis virus (IBV) is a major viral pathogen causing substantial economic losses in the global poultry industry. However, the limited efficacy of commercial vaccines and the absence of approved antiviral drugs underscore the urgent need for novel strategies to combat IBV, particularly in the context of antibiotic‐free poultry production. Emerging evidence suggests that gut microbiota plays a crucial role in shaping host immunity and antiviral resilience. In this study, we demonstrated that gut microbiota composition is a key determinant of IBV resistance in chickens, as revealed through a fecal microbiota transplantation (FMT) model. Utilizing multi‐omics approaches, we conducted a comprehensive characterization of microbiomic, viromic, and metabolic differences among adult chickens, young chickens, and FMT‐treated young chickens for the first time, establishing a mechanistic link between gut microbiota and IBV resistance. Building on these insights, longitudinal microbiome profiling in a newly developed persistent IBV infection model identified *Akkermansia muciniphila* as the key bacterium conferring IBV resistance, with its administration improving survival rates from 30–35% to 90–95%. Additionally, two novel candidate probiotics, *Cloacibacillus porcorum* and *Neglecta timonensis*, exhibited moderate yet measurable protective effects. Mechanistic investigations revealed that *Akkermansia muciniphila* enhances γ‐aminobutyric acid (GABA) production, which suppresses NF‐κB‐driven inflammatory responses, reduces pro‐inflammatory cytokine levels, alleviates nephritis, and upregulates antiviral interferon expression, thereby fortifying host defenses against IBV infection. These findings provide a scientific foundation for deploying live biotherapeutic products as an intervention strategy against IBV and highlight the broader potential of gut microbiota modulation in mitigating infectious diseases and optimizing poultry health management.

## Highlights

- By integrating microbiomics, viromics, and metabolomics, we systematically characterized the gut microbiota in adult and young chickens and constructed their interaction networks for the first time.
- Comparative gut microbiota analysis between adult and young chickens, combined with longitudinal microbiome profiling in a newly established persistent Infectious bronchitis virus (IBV) infection model, identified *Akkermansia muciniphila* (*A. muciniphila*) as a key bacterium conferring resistance to IBV infection.
- *A. muciniphila* enhances IBV resistance by increasing γ‐aminobutyric acid levels, which suppress NF‐κB‐mediated excessive inflammation and promote antiviral interferon responses.
- In addition to *A. muciniphila*, we also identified *Cloacibacillus porcorum* and *Neglecta timonensis* as novel candidate probiotics with the potential to enhance IBV resistance in chickens.

## Graphical abstract

Infectious bronchitis virus (IBV) represents a major threat to poultry production, with current control strategies offering limited protection. In this study, we demonstrate gut microbiome is a critical determinant of IBV resistance using fecal microbiota transplantation. Through an integrated multi‐omics approach and a newly developed persistent IBV infection model, we identify *Akkermansia muciniphila* (*A. muciniphila*) as a key bacterium that enhances resistance to IBV. Administration of *A. muciniphila* significantly improves survival rates in young chickens from 30 to 35% to 90–95%. Mechanistic investigations reveal that *A. muciniphila* enhances γ‐aminobutyric acid (GABA) production, which suppresses NF‐κB‐mediated inflammatory signaling, reduces pro‐inflammatory cytokine levels, mitigates nephritis progression, and upregulates antiviral interferon expression. These findings highlight the translational potential of live biotherapeutic products as a novel strategy for IBV control in poultry production.

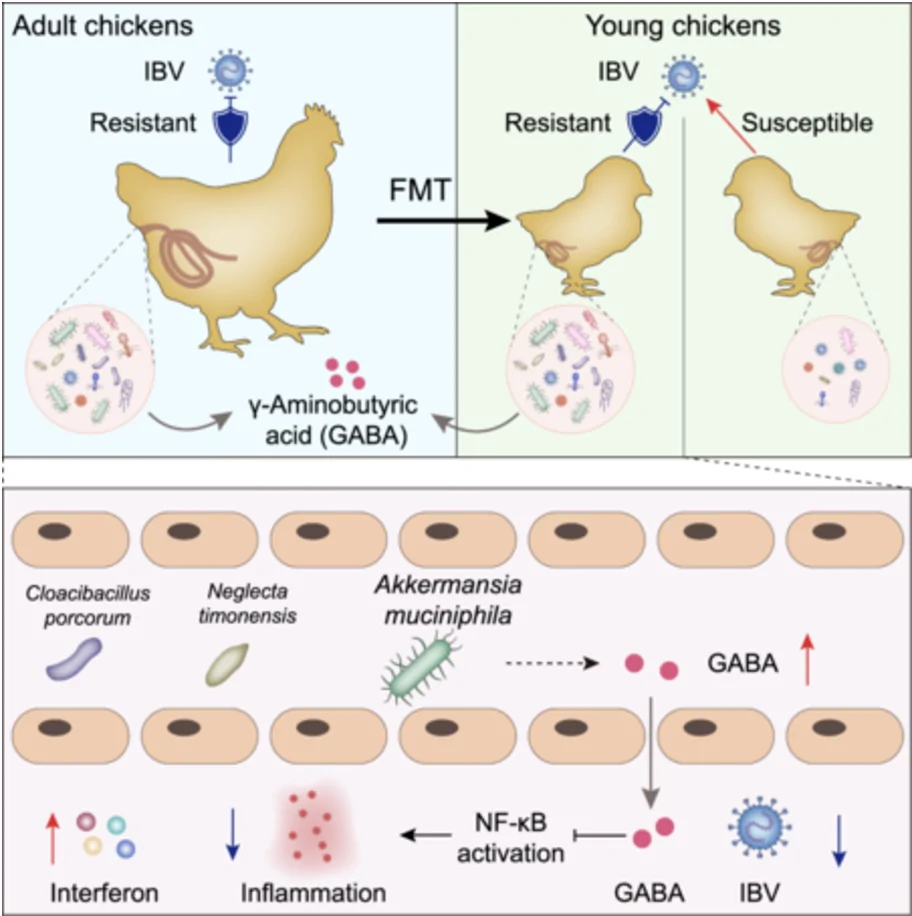

## 1 INTRODUCTION

The gut microbiota plays an essential role in maintaining host health, exerting its influence on host physiology through diverse and multifaceted mechanisms [1, 2]. A key feature of the gut microbiota is its core functional groups, which exist in a dynamic and reciprocal equilibrium with host and environmental factors [3]. Dysbiosis of the gut microbiota has been implicated in the onset and progression of several diseases, including Crohn's disease, type 2 diabetes, colorectal cancer [4, 5, 6, 7], as well as viral infections such as COVID‐19 and influenza [8, 9]. Conversely, deciphering the complex interactions between the gut microbiota and the host has paved the way for novel therapeutic interventions targeting specific microbial populations [10, 11, 12]. For example, microbial metabolites such as short‐chain fatty acids (SCFAs) can enhance the immune responses of CD8^+^ T cells [13] and have the potential to benefit animal bone health [14]. Large‐scale clinical trials have demonstrated that probiotics can restore gut microbiota homeostasis and alleviate post‐COVID‐19 syndrome [15]. These findings underscore the transformative potential of leveraging microbiota–host interactions to develop innovative strategies for preventing and treating diseases with limited control measures.

The gut microbiome has also been linked to the growth performance, development, and overall health of domestic avian species such as chickens and ducks [16, 17]. However, the specific contributions of gut microbiota to the pathogenesis and progression of poultry diseases remain largely unexplored. In China, the yellow‐feathered broilers represent a major poultry breed, with an annual production volume approaching 4 billion birds [18]. Nonetheless, despite their economic importance, these birds remain highly susceptible to infectious bronchitis virus (IBV) [19]. IBV is transmitted primarily through aerosols and primarily affects the respiratory tract. It also exhibits broad tissue tropism, and induces severe disease with high mortality rates in young chickens and reproductive tract damage in adult chickens [19, 20]. As a result, IBV inflicts annual economic losses amounting to millions of dollars and remains one of the most devastating pathogens threatening the global poultry industry [21]. Like other coronaviruses, IBV is characterized by a high mutation rate, strong inflammatory induction, and immune evasion strategies [22, 23, 24]. Moreover, its extensive genetic and serotypic diversity complicates vaccine development, as existing vaccines often fail to provide cross‐protection against emerging variants, leading to frequent immune failures [25, 26, 27]. At present, no approved therapeutics are available for the effective treatment of IBV in clinical settings [28], emphasizing the urgent need for innovative and effective control strategies.

Recent studies suggest that modulating gut microbiota represents a promising avenue for combating viral infections, including those caused by SARS‐CoV‐2 and flaviviruses [15, 29]. Despite these advancements, the role of gut microbiota in mitigating the transmission and infection of IBV remains insufficiently explored. In this study, we applied multi‐omics approaches, integrating microbiomics, viromics, and untargeted metabolomics, to elucidate the gut microbiota‐mediated mechanisms underlying why adult yellow‐feathered chickens (60–70 days) exhibit higher resistance to IBV compared to young yellow‐feathered chickens (10–20 days). Our findings identified *Akkermansia muciniphila* (*A. muciniphila*) as the key microbial species conferring IBV resistance, with *Cloacibacillus porcorum* and *Neglecta timonensis* exhibiting more limited but supportive roles. Using *in vivo* experiments, we demonstrated that *A. muciniphila* significantly improves IBV resistance in young chickens. Furthermore, mechanistic investigation revealed that *A. muciniphila* exerts its protective effects by attenuating inflammation through the gamma‐aminobutyric acid‐nuclear factor kappa B (GABA‐NF‐κB) signaling pathway and stimulating antiviral interferon production, ultimately boosting survival rates. Collectively, these findings provide a mechanistic basis for microbiota‐mediated IBV resistance and introduce a novel live biotherapeutic product (LBPs)‐based strategy for IBV prevention and mitigation, with broader implications for the development of therapeutics against other viral diseases.

## 2 RESULTS

### 2.1 Adult chickens exhibit significantly higher resistance to IBV than young chickens

Based on a decade‐long epidemiological survey conducted by our research team, IBV exhibits a significantly higher mortality rate in younger chickens compared to older ones. Among adult chickens, the mortality rate caused by IBV was observed to be only 4.8%. In contrast, the mortality rate in young chickens reached 29.8% (Figure 1A). The epidemic IBV strains were isolated, propagated, and genotyped, revealing that QX‐like IBV is the most prevalent field strain, accounting for 53.1% of isolates (Table S1). To validate these clinical observations, we established an IBV infection model using the predominant QX‐like genotype strain to infect young (10–20 days old) and adult (60–70 days old) yellow‐feathered broilers. The results revealed that IBV caused a mortality rate of 10% in adult chickens but 60% in younger chickens (Figure 1B).

**Figure 1:**
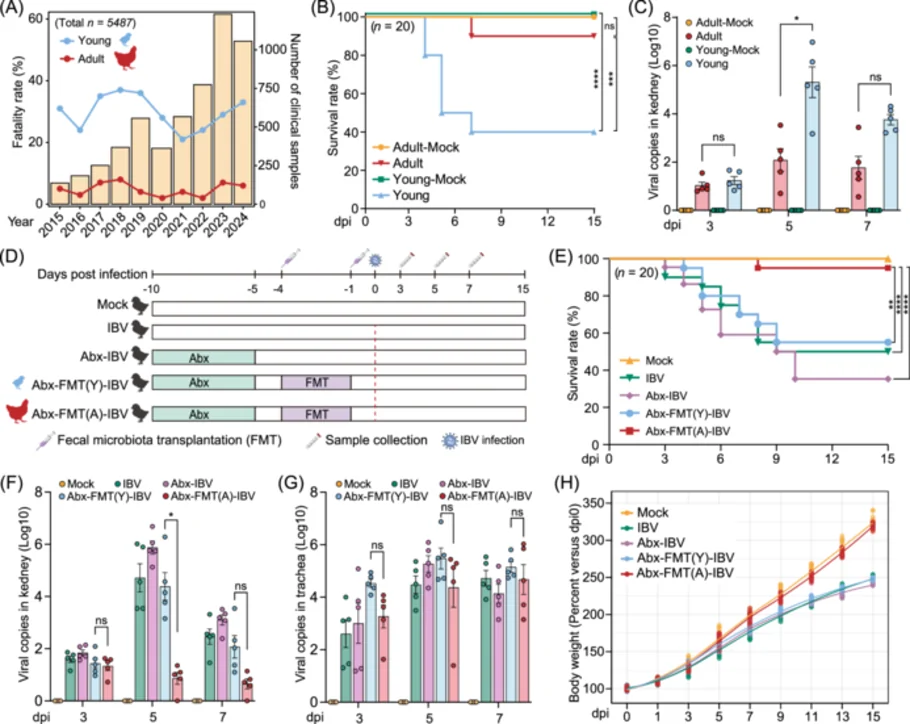
FMT from adult chickens enhances resistance to infectious bronchitis virus (IBV) in young chickens. (A) Epidemiological surveillance data (*n* = 5487) collected by our research team from 2015 to 2024 demonstrate that IBV exhibits higher mortality rates in young chickens within 20 days compared to those of adult chickens older than 60 days. (B) Experimental infection confirms the differential mortality of IBV in chickens at different growth stages (*n* = 20). (C) Viral loads in kidney tissues at 3, 5, and 7 days post infection (dpi) (*n* = 5). (D) Schematic diagram of the FMT experimental workflow. Abx indicates treated with antibiotics, FMT(A) and FMT(Y) indicate fecal microbiota transplantation from adult chickens or young chickens, respectively. (E) Survival curves of chickens in different experimental groups following FMT (*n* = 20). (F) IBV viral loads at Days 3, 5, and 7 dpi in chicken kidney (*n* = 5). (G) IBV viral loads at Days 3, 5, and 7 dpi in chicken trachea (*n* = 5). (H) Body weight changes in chickens across experimental groups. The Kaplan–Meier (K–M) analysis was used to analyze the significance between groups in graphs (B) and (E) (*, **, ***, or **** indicate *p* < 0.05, *p* < 0.01, *p* < 0.001, and *p* < 0.0001, respectively; ns indicates not significant). Bar graphs in (C), (F), and (G) represent the mean ± SEM, with error bars indicating the SEM (* indicates *p* < 0.05; ns indicates not significant). The two‐way ANOVA was used for each comparison.

Reverse transcription quantitative polymerase chain reaction (RT‐qPCR) analysis of viral loads in the kidney, trachea, and ileum on 3, 5, and 7 days post infection (dpi) demonstrated significantly higher viral loads in the kidneys of young chickens compared to adult chickens on 5 dpi (Figure 1C). However, no significant differences were observed in the viral loads of the trachea and ileum (Figure S1A,B). On the 5 dpi, clinical autopsy showed no apparent symptoms in adult chickens, whereas young chickens exhibited hallmark symptoms, including tracheal hemorrhage and mottled kidneys (Figure S1C). Histopathological analysis revealed severe hemorrhage and inflammatory cell infiltration in the kidneys of young chickens post‐infection (Figure S1D), and immunohistochemistry (IHC) analysis demonstrated higher viral loads of the kidney and trachea in young chickens than in adult chickens (Figure S1E). Collectively, these findings corroborate our clinical observations that IBV causes more severe clinical symptoms and higher mortality rates in younger chickens compared to adults.

To investigate whether the differences in IBV‐induced mortality rates between young and adult chickens could be attributed to gut microbiota, we developed a gut microbiota transplantation model (Figure 1D). Fecal microbiota from adult or young chickens were transplanted into 6‐day‐old recipient specific pathogen free (SPF) young chickens pre‐treated with antibiotics for 5 days. The recipient young chickens were subsequently challenged with QX genotype IBV at 10 days of age and monitored for 15 days post‐infection (Figure 1D). The results demonstrated that transplantation of adult chicken fecal microbiota significantly improved the survival rates of recipient young chickens following IBV infection, whereas transplantation of young chicken fecal microbiota had no significant impact on recipient survival (Figure 1E).

Furthermore, the young chickens that receiving adult chicken fecal microbiota exhibited lower viral loads in their trachea, kidney, and ileum compared to those receiving young chicken fecal microbiota (Figure 1F,G; and Figure S2A,B), and the pathological damage induced by IBV in both the kidney and trachea has also been markedly reduced (Figure S2C,D), and the growth performance was also restored (Figure 1H). These findings confirm that gut microbiota can influence IBV resistance in young chickens.

### 2.2 Gut microbiome profiling shows Adult/Young‐FMT chickens share similar microbial structures, distinct from Young chickens

The gut microenvironment is a complex ecosystem comprising diverse bacteria, viruses, and metabolites that collectively regulate host immunity and health [30]. To elucidate the mechanisms underlying the differential effects of the gut microbiota from adult chickens on IBV resistance in young chickens, we employed an integrative approach combining microbiomics, viromics, and untargeted metabolomics to characterize the gut microenvironment across these groups.

16S rRNA gene sequencing was used to detect the chicken gut microbiota. Partial least squares discrimination analysis (PLS‐DA) of microbiomic sequencing data demonstrated robust group clustering and significant divergence in gut microbial composition among adult chickens, young chickens, and young chickens treated with adult chicken fecal microbiota transplantation (young‐FMT) (Figure 2A). Among the 1662 operational taxonomic units (OTUs) identified, 804 were shared across the three groups, whereas 1390 OTUs (83.6%) were shared between adult and young‐FMT chickens, suggesting efficient colonization of adult chicken microbiota in young chicken guts following FMT (Figure 2B; and Figure S3). Alpha diversity analysis further revealed that the gut microbiota of adult and young‐FMT chickens exhibited significantly greater diversity compared to young chickens (Figure 2C,D and Figure S4A,B).

**Figure 2:**
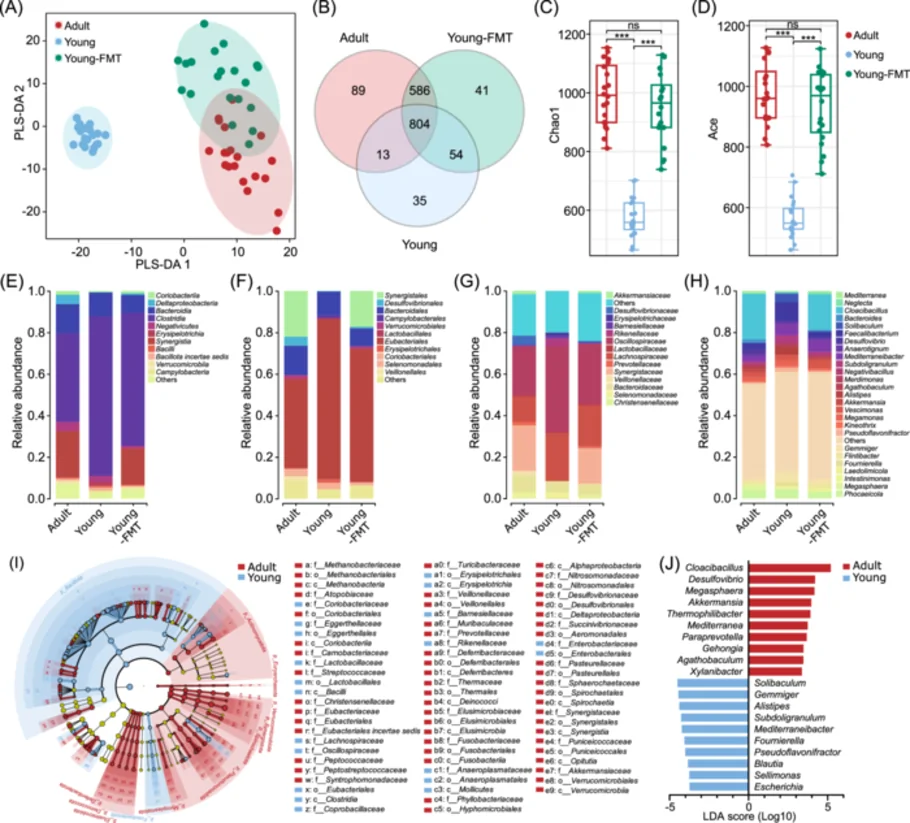
16S rRNA gene sequencing analysis of gut microbiota in adult chickens, young chickens, and FMT‐treated young chickens. (A) Partial least squares discriminant analysis (PLS‐DA) showing differences in gut microbiota composition among the groups (*n* = 20). (B) A Venn diagram illustrating shared and unique operational taxonomic units (OTUs) among the groups. Comparison of alpha diversity indices: Chao1 (C) and Ace (D) among the groups. Gut microbiota composition at the class (E), order (F), family (G), and genus (H) levels across the groups. (I) LEfSe analysis identifying gut microbiota biomarkers distinguishing adult and young chickens. Red and blue circles denote distinct groups, with each circle representing a microbial taxa that plays a key role in the group indicated by its color. The diameter of the circles corresponds to the relative abundance of the respective taxa. Yellow circles represent microbial groups that do not exert significant effects across different groups. From the inner to the outer ring, the circles sequentially represent species classified at the phylum, class, order, family, and genus levels. (J) Top 10 taxa with the highest linear discriminant analysis (LDA) scores differentiating gut microbiota in adult and young chickens. Box and point graphs in (C)–(D) indicate the distribution of the data, the top of the box indicates upper quartile, the bottom of the box indicates the lower quartile, the line in the box indicates median, the top line beyond the box indicates maximum, the bottom line beyond the box indicates minimum (*** indicates *p* < 0.001, ns indicates not significant). The one‐way ANOVA was used for comparison.

Taxonomic classification at the phylum, class, order, family, genus, and species levels revealed substantial differences in microbial composition across three groups (Table S2). The gut microbiota of young‐FMT chickens closely resembled that of adult chickens at nearly all taxonomic levels (Figure 2E–H; and Figure S4C–J). At the class level, young chickens exhibited a predominance of *Clostridia* (76.8%), which was considerably higher than in adult (42.8%) and young‐FMT chickens (64.1%). In contrast, adult and young‐FMT chickens showed significantly higher abundances of *Synergistia* (22.0% and 17.2% vs. <0.1%), *Deltaproteobacteria* (4.5% and 0.9% vs. 0.2%), and *Coriobacteriia* (1.8% and 1.0% vs. 0.5%) (Figure 2E). Linear discriminant analysis effect size (LEfSe) analysis highlighted the top 10 taxa enriched in adult chickens versus those at the genus level, including *Cloacibacillus*, *Desulfovibrio*, *Megasphaera*, *Akkermansia*, and *Thermophilibacter* (Figure 2I,J). The top 10 genera in young‐FMT chickens versus young chickens, including *Cloacibacillus*, *Akkermansia*, and *Mediterranea* (Figure S5A,B). In contrast, the microbiota differences between adult and young‐FMT chickens were minimal (Figure S5C,D).

Functional predictions using PICRUSt2 further revealed distinct microbial metabolic pathway enrichments between adult and young chickens. The gut microbiota of adult chickens was particularly enriched in pathways associated with energy metabolism and digestive system functions (Figure S6 and Table S3). Collectively, these findings provide a comprehensive framework for understanding the unique gut microbial compositions and functional capacities of adult, young, and young‐FMT chickens, emphasizing their roles in differential IBV resistance.

### 2.3 Metatranscriptomic analysis reveals Adult/Young‐FMT chickens share gut virome patterns, diverging from Young chickens

The gut virome, a critical component of the intestinal microenvironment, was analyzed in this study using a metatranscriptomic approach. PLS‐DA results revealed high intra‐group reproducibility and distinct clustering of virome structures among adult chickens, young chickens, and young‐FMT chickens (Figure 3A). Alpha diversity analysis further demonstrated that adult and young‐FMT both harbored a richer virome compared to young chickens, while less significant differences were observed between adult and young‐FMT chickens (Figure 3B–E).

**Figure 3:**
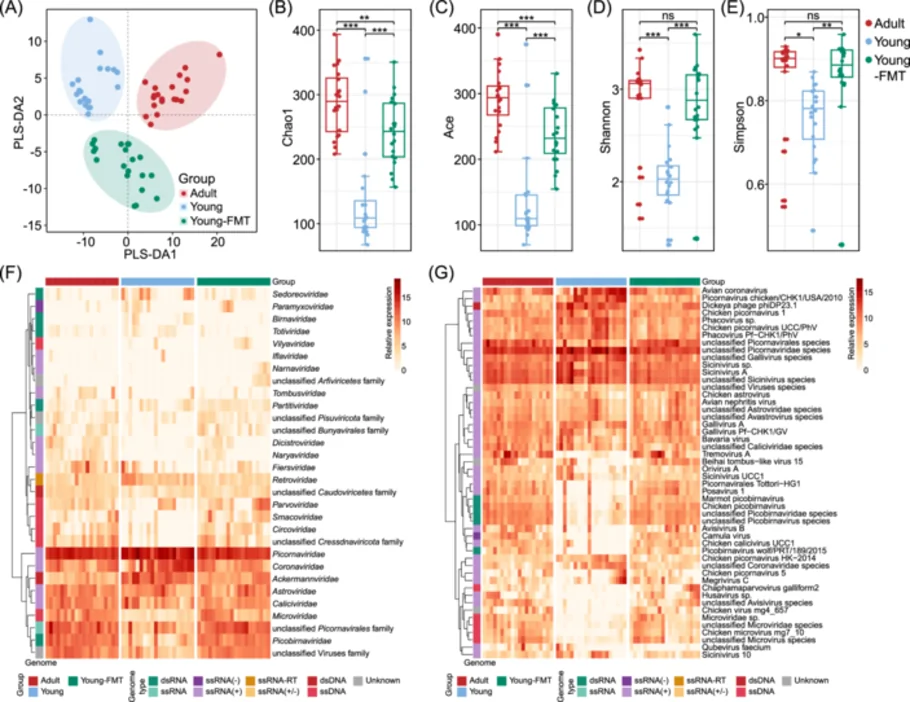
Viromic analysis of gut samples from adult chickens, young chickens, and FMT‐treated young chickens. (A) Partial least squares discriminant analysis (PLS‐DA) showing differences in gut virome composition among the groups (*n* = 20). (B–E) Comparison of alpha diversity indices: Chao1 (B), Ace (C), Shannon (D), and Simpson (E) among the groups. (F) Heatmap showing the relative abundance of viral families across samples. (G) Heatmap showing the relative abundance of viral species across samples. Box and point graphs in (B)–(E) indicate the distribution of the data, the top of the box indicates upper quartile, the bottom of the box indicates the lower quartile, the line in the box indicates median, the top line beyond the box indicates maximum, the bottom line beyond the box indicates minimum (** or *** indicate *p* < 0.01 and *p* < 0.001, respectively; ns indicates not significant). The one‐way ANOVA was used for comparison. FMT, fecal microbiota transplantation.

Taxonomic annotation of sequencing reads revealed substantial differences in viral family abundances. At the phylum level, *Parvoviridae*, *Smacovirida*e, *Circoviridae*, *Microviridae*, and *Picobirnaviridae* were more prevalent in adult and young‐FMT chickens, whereas *Sedoreoviridae*, *Ackermannviridae*, *Coronaviridae*, and *Picornaviridae* dominated in young chickens (Figure 3F). At the species level, adult and young‐FMT chickens showed higher abundances of *Picobirnavirus*, *Tremovirus*, *Avisivirus*, *Posavirus*, and *Microvirus*, while *Gammacoronavirus*, *Gallivirus*, *Rotavirus*, and *Megrivirus* were more abundant in young chickens (Figure 3G). These differences extended across taxonomic ranks, highlighting consistent divergence in the gut virome composition between different ages of chickens (Figure 3F,G, Figure S7A,B and Table S4). Notably, the gut virome profiles of adult and young‐FMT chickens displayed similar patterns, indicating effective virome colonization from adult chicken feces to the recipient young chickens following FMT.

After filtering low‐quality contigs, assembly of viral reads yielded 1083 high‐quality viral contigs, ranging from 1001 to 27,574 nucleotides in length, belonging to families such as *Picornaviridae*, *Coronaviridae*, and *Astroviridae* (Figure S8A). Phylogenetic analysis comparing these contigs with their respective reference genomes revealed significant genetic divergence, suggesting evolutionary adaptations within the gut virome (Figure S8B–D). Assembled DNA viruses were predominantly bacteriophages, which play a potential regulatory role in the gut microbial ecosystem. A total of 51 phage contigs were subjected to host prediction, including members of the families *Microviridae*, *Herelleviridae*, and *Ackermannviridae*. Using the iPHoP software, which integrates BLAST, CRISPR, and iPHoP‐RF algorithms, host prediction identified 13 phage contigs from the *Microviridae* family targeting the genus *Faecalibacterium* and 7 phage contigs from the *Herelleviridae* family targeting the genus *Stercorousia* (Figure S9A–C and Table S5).

Moreover, complex interactions between bacteria and viruses within the gut microenvironment were explored, as these interactions are known to regulate host health and disease outcomes [31]. Interplay networks encompassing bacteria–virus, bacteria–bacteria, and virus–virus interactions were constructed for adult and young chickens, respectively (Figure S10A,B and Table S6). In adult chickens, 2581 interaction pairs were identified, while in young chickens, the number increased to 5551. These findings provide a foundational understanding of the intricate avian gut microenvironment and its potential regulatory mechanisms.

### 2.4 Untargeted metabolomics reveals Adult/Young‐FMT chickens harbor conserved gut metabolic features, divergent from Young chickens

Gut cells and microbiota produce a diverse array of metabolites that play essential physiological roles, shaping a complex and dynamic gut microenvironment in coordination with bacteria and viruses. Untargeted metabolomic analysis identified a total of 3115 gut metabolites in chickens, comprising 523 lipids (16.8%) and 414 amino acids, peptides, and analogs (13.3%) (Figure S11A and Table S7). The primary functions of metabolites include amino acid metabolism, biosynthesis of other secondary metabolites, and lipid metabolism (Figure S11B). PLS‐DA revealed high intra‐group consistency and significant differences in gut metabolite profiles among adult, young, and young‐FMT chickens, with adult and young‐FMT chickens clustering more closely together than young chickens (Figure 4A).

**Figure 4:**
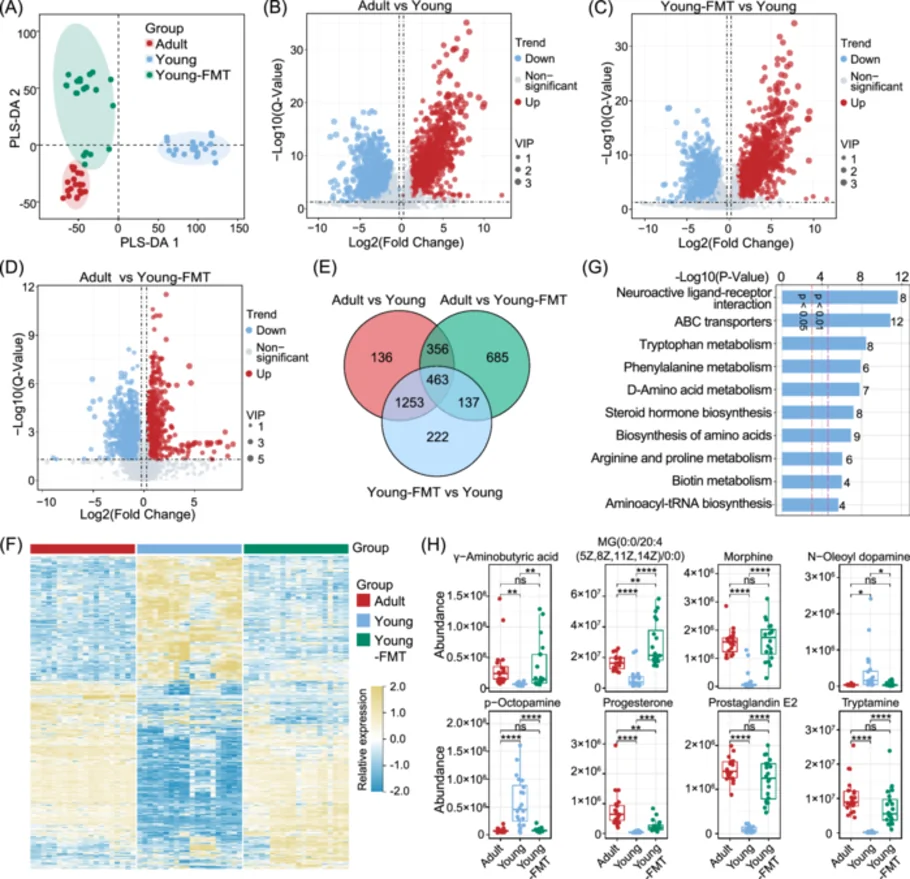
Metabolomic analysis of gut samples from adult chickens, young chickens, and fecal microbiota transplantation (FMT)‐treated young chickens. (A) Partial least squares discriminant analysis (PLS‐DA) showing differences in gut metabolome composition among the groups (*n* = 20). Volcano plots illustrating significantly altered metabolites between adult and young groups (B), between young‐FMT and young groups (C), and between adult and young‐FMT groups (D). VIP indicates Variable Importance in Projection values of the first two principal components from the PLS‐DA model. (E) Venn diagram of differentially expressed metabolites among different comparisons. (F) Heatmap of metabolites relative expression level in each sample. (G) KEGG functions of differentially expressed metabolites between adult and young groups. (H) Abundance of 8 differentially expressed metabolites in neuroactive ligand‐receptor interaction pathway. Box and point graphs in (H) indicate the distribution of the data (*, **, ***, or *** indicate *p* < 0.05, *p* < 0.01, *p* < 0.001, and *p* < 0.0001, respectively; ns indicates not significant). The one‐way ANOVA was used for comparison. KEGG, Kyoto Encyclopedia of Genes and Genomes.

A total of 2239 differentially expressed metabolites were identified in adult chickens compared to young chickens, with 1226 metabolites upregulated and 1013 downregulated. Additionally, the differentially expressed metabolites between young‐FMT and young chickens, as well as adult and young‐FMT chickens, were also identified (Figure 4B–D and Table S8). And the metabolic patterns of adult chickens' gut significantly differ from those of young chickens, but exhibit a more similar trend compared to that of young‐FMT chickens (Figure 4E,F). Kyoto Encyclopedia of Genes and Genomes (KEGG) pathway enrichment analysis revealed that these differentially expressed metabolites between adult and young were enriched in pathways related to neuroactive ligand‐receptor interaction, ABC transporters, and tryptophan metabolism (Figure 4G and Table S9). The expression level of differentially expressed metabolites in the most significant KEGG pathway, neuroactive ligand–receptor interaction, revealed a similar pattern between adult and young‐FMT groups, which is different from Young group (Figure 4H).

Cross‐referencing enriched pathways from metabolomic data with those predicted by microbiome analyses identified 40 shared pathways, highlighting strong concordance between the two datasets (Figure S11C). Correlation analysis using Mantel tests revealed statistically significant associations between gut metabolites and microbial families, such as *Campylobacteraceae* (Mantel's *r* > 0.5 and Mantel's *p* < 0.001) and between the virome and microbial taxa, such as *Clostridiales* and *Peptococcaceae* (Mantel's *r* > 0.25 and Mantel's *p* < 0.01) (Figure S11D).

### 2.5 Low‐dose IBV infection induces gut microbiota shifts, with *Akkermansia* as a key post‐infection biomarker

High‐dose IBV challenge models frequently result in substantial mortality among young chickens, hindering the ability to study longitudinal changes in gut microbiota. To overcome this limitation, a low‐dose infection model was developed to track gut microbiota dynamics over time without inducing fatality. Fecal samples were collected pre‐infection (0) and at 3, 5, and 7 days post‐infection for 16S rRNA gene sequencing (Figure 5A).

**Figure 5:**
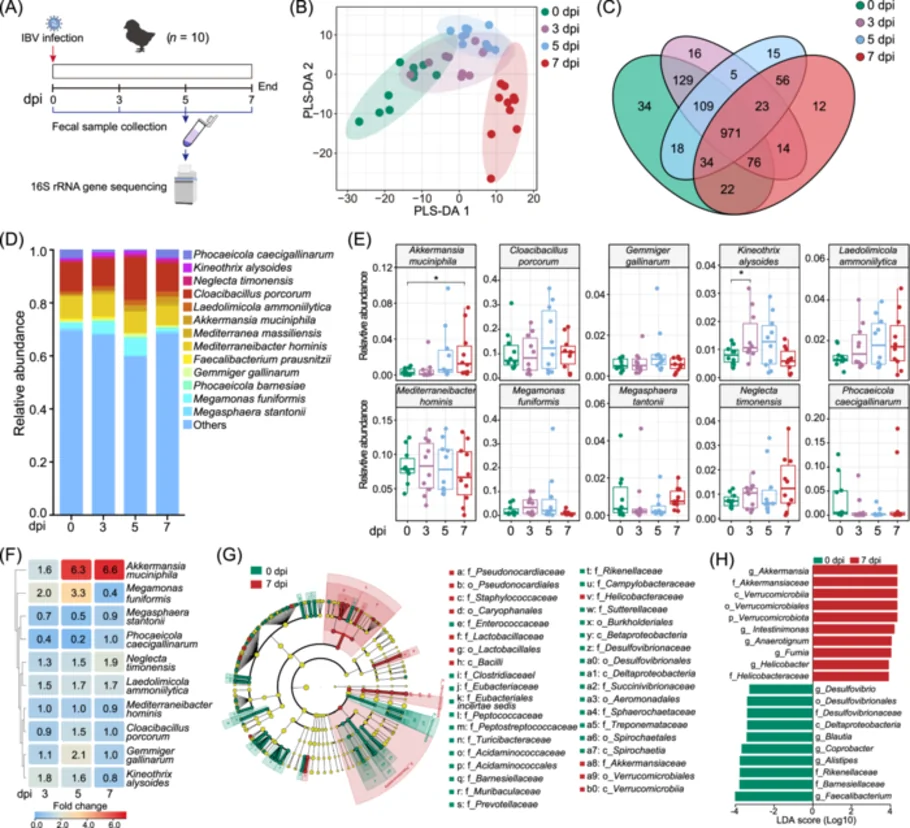
Gut microbiome analysis in a persistent IBV infection model in young chickens. (A) Experimental workflow of the persistent IBV infection model: fecal samples were collected for 16S sequencing prior to IBV infection and at Days 3, 5, and 7 post‐infection. (B) Partial least squares discriminant analysis (PLS‐DA) showing differences in gut microbiota composition across groups (*n* = 10). (C) Venn diagram illustrating shared and unique operational taxonomic units (OTUs) among the groups. (D) Stacked bar plots showing microbiome composition at the species level across groups. (E) Bar plots illustrating abundance changes of the top 10 most abundant microbes before and after IBV infection. (F) Heatmap showing fold changes in the top 10 abundant microbes at 3, 5, and 7 dpi relative to pre‐infection levels. (G) LEfSe analysis identifying significant microbial biomarkers between pre‐infection and Day 7 post‐infection. Red and green circles denote distinct groups, with each circle representing a microbial taxa that plays a key role in the group indicated by its color. The diameter of the circles corresponds to the relative abundance of the respective taxa. Yellow circles represent microbial groups that do not exert significant effects across different groups. From the inner to the outer ring, the circles sequentially represent species classified at the phylum, class, order, family, and genus levels. (H) Top 10 taxa with the highest linear discriminant analysis (LDA) scores differentiating the gut microbiota between pre‐infection and 7 dpi. Box and point graphs in (E) indicate the distribution of the data (* indicates *p* < 0.05, ns indicates not significant). The one‐way ANOVA was used for comparison.

PLS‐DA analysis revealed consistent intra‐group clustering and significant shifts in gut microbial composition following IBV infection (Figure 5B). Across all samples, 971 OTUs were shared between pre‐ and post‐infection groups, reflecting core microbiota retention despite infection (Figure 5C and Table S10). Alpha diversity analysis indicated a transient decline in microbial richness on Days 3 and 5 post‐infection, followed by partial recovery by Day 7 (Figure S12A–D).

At the species level, bacterial abundance exhibited dynamic fluctuations during infection. For instance, *Phocaeicola caecigallinarum* showed an initial decline followed by recovery, whereas *A. muciniphila* displayed a continuous upward trend (Figure 5D). Among the top 10 most abundant taxa, *A. muciniphila* and *Kineothrix alysoides* demonstrated significant increases on Days 7 and 3 post‐infection, respectively (Figure 5E and Figure S12E). Notably, *A. muciniphila* showed the highest fold‐change, with abundance rising 6.3‐fold and 6.6‐fold on Days 5 and 7, respectively (Figure 5F).

LEfSe analysis identified distinct microbial signatures between pre‐infection and Day 7 post‐infection samples, with *Akkermansia* emerging as the most enriched genus on Day 7 (Figure 5G,H). This finding aligns with previous observations that *A. muciniphila* is a key biomarker in the gut microbiota of adult and young‐FMT chickens, where it is significantly more abundant than in young chickens (Figure 2H–J and Figure S4D,J). Additionally, two other species, *Cloacibacillus porcorum* (*C. porcorum*) and *Neglecta timonensis* (*N. timonensis*), were upregulated following IBV infection and exhibited higher abundances in adult and young‐FMT chickens compared to young chickens (Figure S13A–C).

### 2.6 *A. muciniphila* enhances Young chickens' IBV resistance by improving survival and modulating GABA levels

The significant upregulation of *A. muciniphila*, *C. porcorum*, and *N. timonensis* following IBV infection, alongside their higher abundance in the gut microbiota of adult and young‐FMT chickens, prompted the hypothesis that these bacteria contribute to resistance of chickens against IBV. To test this, the three bacterial strains were first propagated. After the safety of these three candidate bacteria was evaluated (Figure S14A,B), each strain was administered individually to young chickens via oral gavage prior to IBV challenge (Figure 6A).

**Figure 6:**
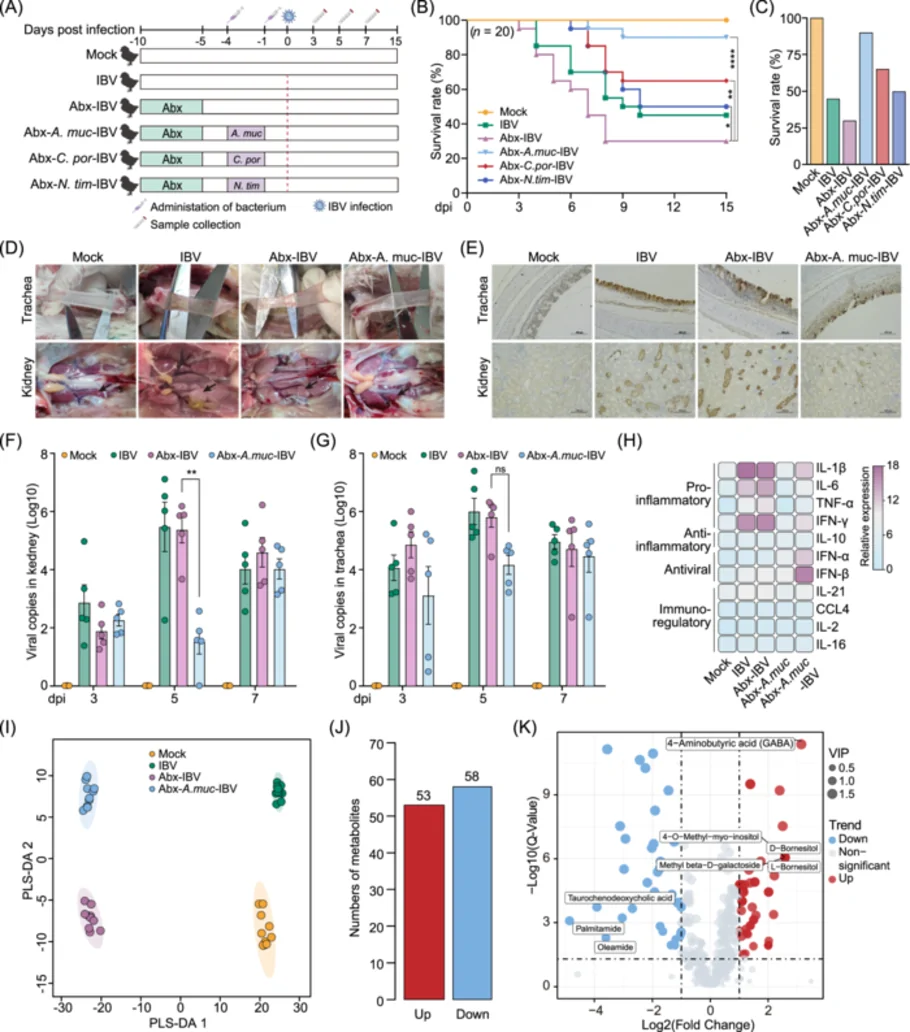
Oral administration of *A. muciniphila* significantly enhances resistance to IBV infection in young chickens. (A) Schematic of the experimental design: young chickens were orally administrated with *C. porcorum*, *N. timonensis*, or *A. muciniphila* followed by IBV challenge. (B) Survival curves of young chickens in different groups (*n* = 20) after IBV challenge. (C) Bar plots summarizing survival rates across groups after IBV challenge. (D) Necropsy images of the trachea and kidneys at 5 dpi (Scale bar, 100 μm), black arrows indicate chicken kidneys. (E) Immunohistochemistry (IHC) analysis of the trachea and kidneys at 5 dpi (Scale bar, 100 μm). Viral loads in the kidney (F) and trachea (G) of young chickens from different groups (*n* = 5) on Days 3, 5, and 7 post‐IBV infection following oral gavage of *A. muciniphila*. (H) Heatmap of cytokine expression levels in blood samples collected on Day 7 post‐IBV infection from young chickens in different groups following oral gavage of *A. muciniphila*. (I) PLS‐DA plot of metabolomic profiles in fecal samples collected on Day 7 post‐IBV infection from young chickens following oral gavage of *A. muciniphila* (*n* = 10). (J) Bar plot of differentially expressed metabolites between the *A. muciniphila*‐administrated group and the IBV‐infected group treated with antibiotics (Abx). (K) Volcano plot of differentially expressed metabolites between the *A. muciniphila*‐administrated group and the Abx‐treated IBV‐infected group. The Kaplan‐Meier (K‐M) analysis was used to analyze the significance between groups in graph (B) (*, **, or *** indicate *p* < 0.05, *p* < 0.01, and *p* < 0.001, respectively). Bar graphs in (D)–(E) represent the mean ± SEM, with error bars indicating the SEM (* indicates *p* < 0.05). The two‐way ANOVA was used for comparison. PLS‐DA, partial least squares‐discriminant analysis.

Among the treatments, *A. muciniphila* markedly improved survival rates of chickens to 90%, compared to 65% for *C. porcorum* and 50% for *N. timonensis* (Figure 6B,C). These results establish *A. muciniphila* as a key factor in conferring resistance to IBV in chickens, with *C. porcorum* and *N. timonensis* providing moderate, yet limited, protective effects.

Given the pronounced efficacy of *A. muciniphila*, subsequent investigations focused on this bacterium. Oral administration of *A. muciniphila* significantly reduced pathological damage and viral loads in the kidneys and trachea of infected young chickens by day 5 post‐infection (Figure 6D–G and Figure S14C).

Serum analysis on 8 days post oral administration of *A. muciniphila* revealed reduced levels of pro‐inflammatory cytokines (IL‐1β, IL‐6, and IFN‐γ) and elevated levels of antiviral interferons (IFN‐α and IFN‐β) in *A. muciniphila*‐treated chickens (Figure 6H). This immune modulation highlights the *A. muciniphila*'s role in reducing inflammation and enhancing antiviral responses.

To further elucidate the underlying mechanism, metabolomic profiling of gut samples collected on Day 5 post‐infection was conducted. The analysis identified 53 upregulated and 58 downregulated metabolites in *A. muciniphila*‐treated and IBV‐infected group compared to the IBV‐infected group (Figure 6I,J, Figure S15A, and Table S11). The KEGG function of differentially expressed metabolites was also enriched (Figure S15B). Among these, GABA exhibited the highest fold increase (Figure 6K). Consistent with prior findings, GABA levels were also significantly higher in the gut of adult chickens compared to young chickens (Figure S16A,B).

GABA, a four‐carbon non‐proteinogenic amino acid, is known for its critical roles in various physiological and biochemical processes across organisms [32]. Notably, GABA has been reported to regulate immune responses, including the inhibition of IL‐1β production [33]. These observations suggest that *A. muciniphila* enhances IBV resistance in young chickens by modulating GABA levels, which subsequently suppress IL‐1β production and mitigate inflammation.

### 2.7 GABA promotes young chickens' survival against IBV by inhibiting NF‐κB‐mediated inflammation

To confirm the role of GABA in enhancing IBV resistance, we administered GABA to young chickens prior to IBV challenge and monitored their survival (Figure 7A). GABA treatment significantly improved chick survival rates following IBV infection (Figure 7B). Serum cytokine analysis on day 5 post‐infection revealed that GABA administration suppressed pro‐inflammatory cytokines (IL‐1β, IL‐6, TNF‐α, and IFN‐γ) while promoting antiviral interferons (IFN‐α and IFN‐β) (Figure 7C–F and Figure S17A,B). GABA also mitigated the pathological damage induced in kidney and trachea, and reduced the viral loads in these tissues (Figure S17C,D).

**Figure 7:**
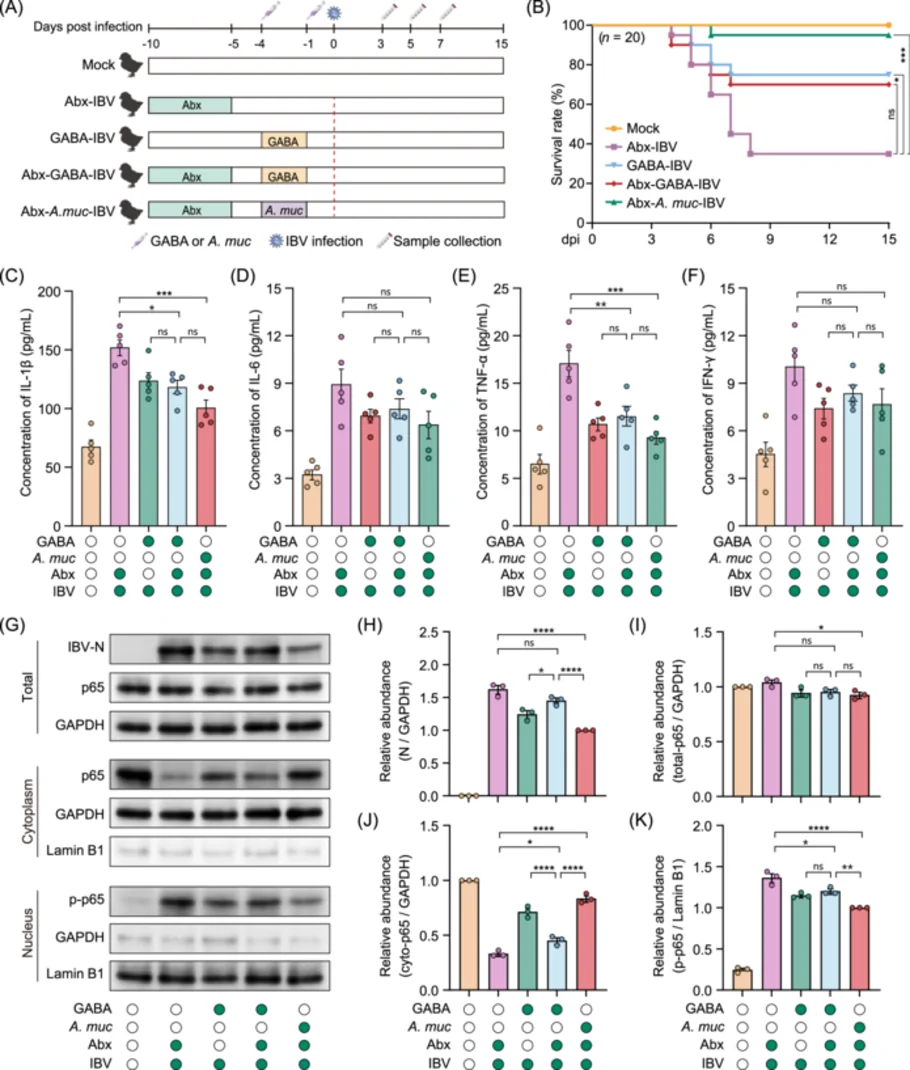
*A. muciniphila* enhances resistance to infectious bronchitis virus (IBV) infection in young chickens through GABA. (A) Schematic diagram of the animal experiment workflow, where young chickens were orally administrated of GABA followed by IBV infection. (B) Survival curves of young chickens in different groups (*n* = 20) following IBV infection. Expression levels of IL‐1β (C), IL‐6 (D), TNF‐α (E), and IFN‐γ (F) in blood samples collected at Day 7 post‐IBV infection using ELISA kits (*n* = 5). Solid circles indicate the groups subjected to the corresponding treatment, while open circles represent those without the corresponding treatment. The same notation applies to other figures thereafter. (G) Western blot analysis of nuclear translocation of p65 in chickens administrated of GABA and *A. muciniphila* following IBV infection. Relative abundance of IBV‐N (H), p65 in total sample (I), p65 in cytoplasm sample (J), and p‐p65 in nucleus sample (K) (*n* = 3). The Kaplan–Meier (K–M) analysis was used to analyze the significance between groups in graph (B) (* or *** indicate *p* < 0.05 and *p* < 0.001, respectively). Bar graphs in (C)–(F) and (H)–(K) represent the mean ± SEM, with error bars indicating the SEM (**, ***, or **** indicate *p* < 0.01, *p* < 0.001, and *p* < 0.0001, respectively). The one‐way ANOVA was used for comparison.

IBV infection induces inflammation and tissue damage via activation of the NF‐κB signaling pathway [34]. GABA has been shown to inhibit inflammation by reducing IL‐1β expression [33]. To investigate whether the anti‐inflammatory effects of GABA are mediated through NF‐κB signaling, we examined p65 protein expression in the kidneys of infected young chickens on 5 dpi. GABA treatment significantly suppressed IBV‐induced p65 phosphorylation and nuclear translocation, thereby mitigating NF‐κB‐mediated inflammatory responses and kidney tissue damage (Figure 7G–K).

These findings confirm that GABA plays a pivotal role in regulating immune responses and inflammation during IBV infection, primarily by modulating the NF‐κB signaling pathway.

## 3 DISCUSSION

A decade of clinical observations reveals that adult chickens exhibit stronger resistance to IBV than young chickens, but the role of the gut microbiome remains unclear. Given its crucial role in poultry development and health [35, 36] and the industry's shift toward antibiotic‐free farming [37, 38], understanding the role of gut microbiome in IBV resistance is of both scientific and practical significance.

IBV is one of the most significant viral pathogens affecting the global poultry industry, causing millions of dollars in economic losses due to its high prevalence, broad tissue tropism, and severe morbidity and mortality rates [21]. IBV is one of the most significant viral pathogens affecting the global poultry industry, causing millions of dollars in economic losses due to its high prevalence, broad tissue tropism, and severe morbidity and mortality rates [39]. However, their efficacy is limited by inadequate cross‐protection against diverse IBV variants [39, 40]. Moreover, the absence of approved antiviral drugs, coupled with restrictions on drug use in the poultry industry, underscores the need for alternative strategies that align with antibiotic‐free and sustainable farming policies. While previous studies have demonstrated the critical role of gut microbiota in IBV resistance [41], its potential as a therapeutic intervention remains unclear. Here, we confirm that FMT from adult chickens significantly improves the survival rate of young chickens following IBV infection (Figure 1D,E), a crucial finding given that IBV causes the most severe damage in young birds [39]. Additionally, FMT reduces viral loads (Figure 1F,G and Figure S2D) and mitigates tissue damage in the kidneys and trachea (Figure S2B,C), the primary target organs of IBV [42]. FMT has been demonstrated as a promising strategy for significantly enhancing antioxidant capacity, immune function, and intestinal glucose transport in young chickens [35]. Our findings highlight FMT as a promising microbiome‐based therapeutic strategy for IBV control and a sustainable alternative for poultry disease management in the context of antibiotic‐free farming.

By integrating multi‐omics analyses, we comprehensively characterized the gut microbiome of adult chickens, young chickens, and young chickens treated with FMT. Compared to young chickens, young‐FMT chickens exhibited a significant decrease in *Bacillota* and an increase in *Synergistota*, mirroring the microbial composition of adult donor chickens (Figure S4C). This shift indicates the successful colonization of transplanted microbiota. Previous studies have similarly reported that FMT reshapes the gut microbiome of recipient chickens, aligning it closely with that of donors [43]. However, while FMT transfers not only bacteria but also viruses and metabolites, little research has explored these additional components. In this study, we further examined viromic and metabolic profiles post‐FMT and found that young‐FMT chickens exhibited viral (Figure 3F,G) and metabolic (Figure 4F) compositions similar to those of adult donors, distinguishing them from untreated young chickens. While prior research has shown that FMT alters the metabolome of recipients, it has not directly compared these changes with donor metabolic profiles [44, 45]. Our results suggest that FMT facilitates not only beneficial bacterial colonization but also the transfer of advantageous metabolites, such as GABA (Figure 4H). However, we also observed an increased relative abundance of IBV in young‐FMT chickens compared to untreated young chickens, potentially due to viral transmission from adult donors (Figure 3G). This concern aligns with warnings from the U.S. Food and Drug Administration (FDA) regarding the risk of transmitting SARS‐CoV‐2 during FMT procedures [46]. Therefore, to ensure the safety of FMT‐based interventions in poultry, stringent screening of FMT donors for pathogenic viruses is essential to mitigate potential transmission risks.

In a newly established persistent IBV infection model, we identified three candidate probiotics, *A. muciniphila*, *C. porcorum*, and *N. timonensis*, and validated their roles in enhancing IBV resistance in chickens. Notably, administration of *A. muciniphila* significantly improved chick survival to 90–95%, whereas the other two bacteria exhibited relatively modest effects (Figure 6B,C). Among microbial species, *A. muciniphila* emerged as the most promising, given its well‐documented benefits in gut health, aging mitigation, and metabolic regulation [47, 48, 49, 50, 51, 52]. Recent studies have shown its antiviral potential against *Bunyaviridae* in mice [53], and our findings extend its therapeutic scope to gamma‐coronavirus infections, demonstrating its potential for improving poultry disease resistance. *A. muciniphila* shows promise as a poultry probiotic, but its large‐scale production is challenged by oxygen sensitivity and reliance on animal‐derived growth substrates [54]. For commercial poultry farming, further optimizing these processes to reduce costs is critical to align with industry efficiency demands. Recent advances offer possible solutions, such as genome‐scale metabolic models identifying mucin‐derived monosaccharides as optimal substrates and scalable anaerobic workflows for viable cell preparation [55]. Storage stability is critical for the industrial application of *A. muciniphila*, and spray‐drying has emerged as a cost‐effective, scalable encapsulation strategy. With optimized matrix and temperature, this approach maintains robust aerobic viability and effectively mitigates core barriers to the distribution and shelf‐life sustainability of *A. muciniphila* [56]. Host‐sourced probiotics typically have superior gut colonization efficiency due to pre‐adaptation to the host's physiological environment. Therefore, the environmental adaptability of *A. muciniphila* can be enhanced by isolating strains from the target host. Furthermore, pasteurized *A. muciniphila* or outer membrane proteins such as Amuc_1100 and Amuc_1409 retain biological activity, which offers potential for developing more cost‐effective or storage‐stable feed additives [57]. Given the growing resistance to traditional antiviral drugs, probiotic‐based strategies present a viable alternative, offering safety, cost‐effectiveness, and immunomodulatory advantages [58, 59]. Our study lays the groundwork for leveraging *A. muciniphila* and other beneficial bacteria in poultry farming and highlights the potential of multi‐strain probiotic formulations to enhance therapeutic efficacy [60]. Resolving the remaining practical barriers will be critical to fully realizing the translational potential of *A. muciniphila* as a poultry health intervention.

IBV infection induces severe inflammation in the kidneys and trachea, leading to high mortality in chickens [61]. Excessive inflammation is also a hallmark of coronavirus infections, including SARS‐CoV‐2, where cytokine storms drive severe outcomes [62]. Controlling inflammation is therefore a key strategy in mitigating disease severity. The gut microbiota plays a crucial role in regulating host inflammation and immunity via direct and indirect mechanisms [63, 64, 65, 66]. For instance, extracellular vesicles from *Roseburia intestinalis* suppress inflammation and improve gut health [64]. Similarly, *A. muciniphila* modulates host immunity through extracellular vesicles and other pathways [67, 68]. Previous studies have shown that its membrane phosphatidylethanolamine (PE) influences cytokine secretion and dendritic cell activation, fine‐tuning immune responses [69]. Our study demonstrated that *A. muciniphila* enhances IBV resistance by increasing GABA levels, which in turn suppresses pro‐inflammatory cytokines and alleviates kidney inflammation (Figure 7G). GABA is a widely distributed non‐proteinogenic amino acid that functions as a neurotransmitter and a modulator of immune and metabolic processes [33, 70, 71]. GABA can be derived from multiple sources. It is primarily produced by GABAergic neurons [72] and can also be secreted by non‐neuronal cells such as B cells [73], cancer cells [74], and members of the microbiota [75]. Increasing evidence indicates that GABA interacts with the cellular immune system to regulate immune responses. For example, GABA binds to GABA receptors on macrophages to promote the polarization of tumor‐associated macrophages toward the M2 phenotype [76]. Additionally, microbially derived GABA has been shown to modulate cell proliferation, differentiation, and apoptosis, while enhancing immune function by increasing the expression of avian β‐defensins in the chicken gut [75], thereby establishing functional connections between the gut microbiota and the host. GABA also could inhibit IL‐1β expression in macrophages by modulating NF‐κB signaling and inflammasomes [33]. In IBV‐infected young chickens, NF‐κB‐mediated inflammation was excessively activated, but *A. muciniphila*‐induced GABA effectively suppressed this response (Figure 7G–K). Despite these observations, the specific origin of the increased GABA levels following *A. muciniphila* administration and the precise mechanisms by which GABA modulates inflammatory and immune responses in IBV‐infected chickens remain to be fully elucidated. Future research will focus on these unresolved questions to thoroughly elucidate the *A. muciniphila*‐GABA‐immune axis in IBV resistance.

This study leveraged multi‐omics approaches, integrating microbiomics and metabolomics, to explain the observed differential IBV resistance between young chickens and adult chickens. We identified *A. muciniphila* as a key functional member of the gut microbiota responsible for IBV resistance. Mechanistically, we demonstrated that *A. muciniphila* enhances resistance by upregulating GABA, thereby suppressing NF‐κB‐driven inflammation, mitigating nephritis, and strengthening antiviral immunity. These findings offer novel insights into microbiota‐mediated antiviral defense and establish a scientific framework for advancing microbiome‐targeted LBPs as interventions against IBV and other emergent viral pathogens.

Nevertheless, this study has certain limitations. First, our findings are based on yellow‐feathered broilers, and further validation in other commercially important chicken lines, such as white‐feathered broilers, is needed. Second, IBV is highly diverse, with at least eight circulating genotypes. Our study focused on the predominant QX‐like genotype, and whether *A. muciniphila* confers protection against other variants remains to be determined. Additionally, future studies should assess whether combining *A. muciniphila* with *C. porcorum*, *N. timonensis*, or a multi‐strain probiotic formulation could enhance IBV resistance. Addressing these gaps will help refine probiotic applications across different poultry populations and viral strains.

## 4 CONCLUSION

Our study provides novel insights into how *A. muciniphila* confers IBV resistance via GABA‐mediated immunomodulation, offering a new paradigm for microbiota‐driven antiviral strategies in poultry. These findings pave the way for microbiome‐based interventions. Future research should focus on refining probiotic applications, optimizing multi‐strain formulations, and further elucidating microbiota‐metabolite interactions in viral disease resistance. As antibiotic‐free farming continues to gain traction, FMT‐based biological control strategies hold immense promise for sustainable poultry health management and disease prevention.

## 5 MATERIALS AND METHODS

### 5.1 Clinical samples and experimental animals

Clinical samples from diseased chickens were obtained from poultry farm owners cooperating with Wen's Food Group and Harbin Veterinary Research Institute (HVRI). Samples, including trachea, kidneys, and other diseased tissues, were collected from diseased or dead chickens and stored at −80°C for IBV detection. For in vivo experiments, yellow‐feathered broilers were purchased from collaborating farmers of Wens Food Group, and SPF chickens were obtained from Guangdong Wens Dahuanong Biotechnology Co., Ltd.

### 5.2 Reverse transcription quantitative polymerase chain reaction (RT‐qPCR)

The tissue samples of chickens were first ground with a tissue grinder, then the supernatant was used to extract RNA using TRIzol Reagent (Invitrogen Life Technologies) and reverse transcribed using a ReverTra Ace qPCR RT Master Mix with gDNA Remover kit (TOYOBO). Relative quantitative PCR was performed for the detection of the N gene [77] using Hieff qPCR SYBR Green Master Mix (Yeasen) on a Light‐Cycler 480 PCR system (Roche), and the GAPDH gene was used as the reference gene for normalization. The cycling conditions were as follows: 95°C for 5 min, followed by 40 cycles of 95°C for 10 s, 58°C for 10 s, and 72°C for 30 s, with subsequent incubations at 95°C for 5 s, 60°C for 1 min, and 95°C for 15 s. Data were analyzed using the 2^−ΔΔCT^ method and presented as the change (n‐fold) relative to either the control group inoculated with PBS or non‐infected cells.

### 5.3 Infectious bronchitis virus (IBV) propagation

The QX‐type IBV strain H24 was isolated and stored in our laboratory. IBV was propagated in 9‐ to 11‐day‐old SPF embryonated chicken eggs at 37°C with 65% humidity. Inoculated eggs were monitored daily by candling for embryonic mortality. After 48 h, the allantoic fluid was harvested, and the virus titer was determined using the 50% egg infective dose (EID_50_) method as described previously [78].

### 5.4 Establishment of IBV infection models in adult and young chickens

Eighty untreated 10‐day‐old and eighty untreated 60‐day‐old yellow‐feathered broilers were each randomly divided into two groups with 40 birds per group, forming the adult control group (Adult‐Mock), adult challenge group (Adult), young chicken control group (Young‐Mock), and young chicken challenge group (Young). Birds in the challenge groups were inoculated with IBV at a concentration of 1 × 10⁵ EID₅₀/mL, with 0.2 mL administered per bird via the oculonasal route, while birds in the control groups received an equal volume of physiological saline. Throughout the experiment, SPF chickens were housed in isolators; a heat lamp was used in the early stage, and the temperature was gradually reduced to room temperature thereafter. The survival status of the birds was observed daily, and sufficient feed was provided to allow ad libitum feeding and drinking.

### 5.5 Animal experimental design for IBV infection after chicken intestinal microbiota transplantation

Two hundred 1‐day‐old male SPF chickens were randomly divided into five groups with 40 birds per group, and the grouping and animal treatment procedures are illustrated in Figure 1D. For the control group (Mock group), SPF chickens were given physiological saline continuously for 5 days, followed by administration of 0.2 mL physiological saline via the oculonasal route on the 8th day. In the IBV challenge group (IBV group), SPF chickens were given physiological saline continuously, and then 0.2 mL IBV virus solution was administered via the oculonasal route on the 8th day. For the antibiotic (Abx group)‐treated and IBV‐challenged group (Abx‐IBV group), SPF chickens were given physiological saline containing antibiotics continuously, followed by administration of 0.2 mL IBV virus solution via the oculonasal route on the 8th day. In the young chicken intestinal microbiota transplantation group [Abx‐FMT(Y)‐IBV group], SPF chickens were given physiological saline containing antibiotics continuously, then gavaged with cecal contents from young chickens, and subsequently challenged with IBV. For the adult chicken intestinal microbiota transplantation group [Abx‐FMT(A)‐IBV group], SPF chickens were given physiological saline containing antibiotics continuously, then gavaged with cecal contents from adult chickens, and subsequently challenged with IBV.

To maximize the potential colonization of donor microbiota, the intestinal microbiota of recipient chickens was depleted through the administration of an antibiotic cocktail. Each SPF chicken was provided with drinking water containing a mixed antibiotic combination consisting of 0.25 g/L vancomycin, 10 g/L neomycin, 10 g/L metronidazole, 0.1 g/L amphotericin B, and 1 g/L ampicillin. The antibiotic treatment lasted for 4 days, with fresh antibiotic‐containing drinking water replaced every 2 days. On the 5th day, SPF chickens were given normal drinking water, and intestinal microbiota transplantation was performed 24 h later.

Sixty 10‐day‐old and sixty 60‐day‐old male yellow‐feathered broilers used as donor chickens were sourced from free‐range poultry farms of farmers collaborating with Wens Foodstuff Group, Guangdong Province. All donor chickens underwent strict screening before use, visual inspection was conducted 24 h before sacrifice to exclude individuals with excessively weak phenotypes such as emaciated bodies and slow movement or overly strong phenotypes such as abnormally large body sizes and aggressive behavior, ensuring the selected donors had similar growth status; all donor chickens were monitored continuously for five consecutive days to confirm the absence of signs including diarrhea, lethargy, ruffled feathers, respiratory distress like sneezing and wheezing, and reduced feed or water intake; RT‐qPCR was also used to detect four common pathogenic viruses that may disrupt gut microbiota or affect experimental outcomes, including IBV, newcastle disease virus, avian influenza virus (AIV, H9 subtype), and infectious bursal disease virus.

After sacrifice, cecal contents were collected from each donor chicken under sterile conditions using autoclaved instruments and sterile collection tubes to avoid exogenous contamination. The collected cecal contents were immediately subjected to quality screening. Only samples with normal physiological characteristics, brown to yellowish‐brown color, well‐formed consistency, and no presence of mucus, blood, pus, or foreign debris were selected, excluding those with abnormal appearance or signs of pathological changes. Each qualified sample was collected at a volume approximately equivalent to the size of two soybeans per bird. To maintain microbial viability, all cecal content samples were processed within 30 min after collection to minimize microbiota degradation.

Cecal contents from three donor chickens of the same age group were first pooled, and half of this pooled mixture was exclusively used for FMT in one recipient SPF chicken, with the recipient assigned to either the Abx‐FMT(Y)‐IBV or Abx‐FMT(A)‐IBV group based on the age of the donor pool. The remaining half of the pooled cecal contents from these three donors was retained for microbiome sequencing to ensure sufficient sample quantity for subsequent sequencing analyses. The microbiota suspension used for FMT was prepared under anaerobic conditions following the protocol described previously [79]. Briefly, the cecal contents intended for FMT were placed in 50 mL sterile centrifuge tubes, an equal volume of sterile physiological saline was added, and the mixture was thoroughly mixed before being filtered through a 60‐mesh stainless steel cell sieve. The filtered turbid solution was gavaged into SPF chickens in the transplantation groups using a gavage needle. SPF chickens in the antibiotic‐pretreated transplantation groups were gavaged with the solution at a dose of 100 μL per bird on the 6th and 9th days, respectively.

The propagated virus solution was diluted in advance with sterile physiological saline to a concentration of 5 × 10⁵ EID₅₀/mL for challenging SPF chickens in the challenge groups. The challenge was administered via the oculonasal route, with a dose of 0.2 mL per chicken.

### 5.6 Collection of intestinal samples for multi‐omics analysis

To analyze the differences in intestinal microenvironment among adult chickens (Adult group), young chickens (Young group), and young chickens transplanted with adult chicken microbiota (Young‐FMT group), fresh cecal content samples were collected from 60 adult chickens (Adult), 60 young chickens (Young), and 60 SPF chickens (Young‐FMT group) that had been transplanted with intestinal microbiota from the same batch of adult chickens using the aforementioned method. Samples in each group were collected from three chickens to reduce individual variation, resulting in 20 replicate samples per group. After thorough mixing, the samples were immediately frozen in liquid nitrogen and stored at −80°C for 16S rRNA gene sequencing, viromics sequencing, and untargeted metabolomics sequencing.

### 5.7 Establishment of a low‐dose IBV infection model in young chickens

Ten birds were randomly selected from 50 untreated 10‐day‐old yellow‐feathered broilers, sacrificed, and their cecal contents were collected for 16S rRNA sequencing. The remaining birds were challenged with a low dose of IBV, with the virus administered at a concentration of 1 × 10^3^ EID₅₀/mL and a volume of 0.2 mL per bird via the oculonasal route. Throughout the experiment, all chickens were housed in isolators; a heat lamp was used in the early stage, and the temperature was gradually reduced to room temperature thereafter. The survival status of the birds was observed daily, and sufficient feed was provided to allow ad libitum feeding and drinking.

### 5.8 Preparation of candidate transplanted bacterial strains and evaluation of the safety in chickens

The probiotic strains used in this study included *Akkermansia muciniphila* ATCC BAA‐835 (purchased from the American Type Culture Collection, ATCC), *Cloacibacillus porcorum* DSM 25858 (purchased from the Leibniz Institute DSMZ‐German Collection of Microorganisms and Cell Cultures, DSMZ), and *Neglecta timonensis* DSM 102082 (purchased from DSMZ). All strains were subjected to activation and propagation under species‐specific optimal conditions to ensure viability and purity.

Strain activation was performed in an anaerobic workstation. Glycerol stocks of each strain were thawed, and 50–100 μL of the bacterial suspension was inoculated into 5–6 mL of species‐specific liquid medium. The mixture was gently homogenized, and 500 μL of the inoculated medium was spread onto the corresponding solid agar plates. The remaining liquid culture was incubated at 37°C until reaching the logarithmic growth phase, and strain identity was verified by 16S rRNA gene sequencing before further propagation or cryopreservation. The solid agar plates were placed in anaerobic bags with gas‐generating packets, sealed tightly, and incubated in the dark at 37°C for 72 h. After incubation, single colonies with consistent morphology were observed, confirming strain purity and the absence of contamination. For long‐term storage, the cultured bacterial suspension was mixed with 20% glycerol at a 1:1 ratio under anaerobic conditions and stored at −80°C.

*A. muciniphila* was streaked onto brain‐heart infusion (BHI) agar supplemented with 0.5% porcine mucin and 0.05% cysteine in an anaerobic workstation, followed by incubation in an anaerobic jar at 37°C for 48 h [80]. *Cloacibacillus porcorum* was cultured in brain–heart infusion broth supplemented with 0.05% cysteine, 0.0001% resazurin, 20 mM arginine, and 20 mM histidine (BHIAH medium) at 39°C for 72 h [81]. *Neglecta timonensis* was grown on Columbia agar enriched with 5% sheep blood under anaerobic conditions at 37°C for 72 h [82].

After cultivation, bacterial cultures were centrifuged at 4000×*g* for 10 min, washed twice with sterile phosphate‐buffered saline (PBS), and resuspended in 20% glycerol/PBS to a final concentration of 5 × 10^9^ CFU/mL. Each chicken in the probiotic treatment groups received 200 μL of the bacterial suspension per administration via oral gavage, corresponding to a single dose of 1 × 10^9^ CFU per chicken. The dosage was determined based on prior poultry probiotic research and preliminary experiments to balance biological activity and host safety, ensuring consistent administration across all treatment groups for direct efficacy comparison.

Fifty 1‐day‐old male SPF chickens were randomly divided into five groups with 10 birds per group, including three bacterium‐administered groups (Abx‐*A. muc* group, Abx‐*C. por* group, Abx‐*N. tim* group), a Mock group, and an Abx group. The treatment procedures for the chickens were conducted under the same conditions as the subsequent bacterium function verification experiment. Throughout this pre‐experiment, SPF chickens were housed in isolators; a heat lamp was used in the early stage, and the temperature was gradually reduced to room temperature thereafter. A standard diet was provided to allow ad libitum feeding and drinking.

After 5 days of antibiotic pretreatment, a 24‐h interval was allowed, and then the chickens in the Abx‐*A. muc*, Abx‐*C. por*, and Abx‐*N. tim* were gavaged with *Akkermansia muciniphila*, *Clostridium porcorum*, and *Neisseria timonensis*, respectively, on the 6th and 9th days. Each gavage dose was 1 × 10⁹ CFU per chicken each time. The Mock group and Abx group did not receive bacterial gavage, and none of the five groups were challenged with IBV.

During the entire experimental period, SPF chickens were continuously housed in isolators, with a heat lamp used in the early stage and the temperature gradually reduced to room temperature thereafter. A standard diet was provided, and free feeding and drinking were allowed after the cessation of antibiotic administration. The survival status of chickens in each group was observed and recorded throughout the pre‐experiment to evaluate the safety of the three bacteria.

### 5.9 Experimental design for bacterium function verification

One hundred and twenty 1‐day‐old male SPF chickens were randomly divided into six groups with 20 birds per group, and the treatment procedures for the chickens are illustrated in Figure 6A. Throughout the experiment, SPF chickens were housed in isolators. Heat lamps were used in the early stage, and the temperature was gradually reduced to room temperature thereafter. A standard diet was provided to allow free feeding and drinking. After 5 days of antibiotic pretreatment, a 24‐h interval was allowed, and then the chickens were gavaged with *Akkermansia muciniphila*, *Clostridium porcorum*, and *Neisseria timonensis* on the 6th and 9th days, respectively. Each gavage dose was 1 × 10^9^ CFU per chicken each time, as described above. The propagated virus solution was diluted in advance with sterile physiological saline to a concentration of 5 × 10⁵ EID₅₀/mL for challenging. On the 10th day, the challenge was administered via the oculonasal route, with a dose of 0.2 mL per chicken. During the entire experiment period, SPF chickens were housed in isolators, a heat lamp was used in the early stage with the temperature gradually reduced to room temperature thereafter, a standard diet was provided, and ad libitum feeding and drinking were allowed after the cessation of antibiotic administration.

### 5.10 Animal experimental design for oral GABA administration

Two hundred 1‐day‐old SPF chickens were randomly divided into five groups with 40 birds per group, and the grouping and animal treatment procedures are illustrated in Figure 7A. The first group was the Mock group (control group), the second was the Abx‐IBV group (antibiotic‐treated followed by IBV challenge), the third was the GABA‐IBV group (GABA‐gavaged followed by IBV challenge), the fourth was the Abx‐GABA‐IBV group (antibiotic‐treated, then GABA‐gavaged, followed by IBV challenge), and the fifth was the Abx‐A.muc‐IBV group (antibiotic‐treated, then *Akkermansia muciniphila*‐gavaged, followed by IBV challenge). During the entire experiment period, SPF chickens were housed in isolators, a heat lamp was used in the early stage with the temperature gradually reduced to room temperature thereafter, a standard diet was provided, and ad libitum feeding and drinking were allowed after the cessation of antibiotic administration.

### 5.11 DNA extraction and quality assessment

DNA was extracted from 200 mg fecal samples using the MagPure Stool DNA KF Kit B (MAGEN, Guangzhou, China) following the manufacturer's instructions, with nucleic acid purification system Kingfisher (Thermo Fisher). After extraction, the concentration of DNA samples was quantified using the Qubit™ dsDNA HS Assay Kit (Thermo Fisher ScientificTM), and their integrity was verified via 0.8% agarose gel electrophoresis with 3% SYBR Safe DNA Gel Stain for visualization.

### 5.12 Library preparation and 16S rRNA sequencing

Library preparation was initiated with PCR amplification using the 2 × Phanta Max Master Mix (VAZYME), where the V3–V4 hypervariable regions of the bacterial 16S rRNA gene were targeted. Amplification was performed with the degenerate forward primer 338F (5'‐ACTCCTACGGGAGGCAGCAG‐3') and reverse primer 806R (5'‐GGACTACHVGGGTWTCTAAT‐3'). Subsequent purification of PCR amplicons was carried out using DNA magnetic beads (BGI) to remove non‐specific products and residual reagents. After purification, double‐stranded libraries were denatured to single strands, circularized into single‐stranded circular DNA, and residual linear single strands were digested to avoid interference. The single‐stranded circular library was amplified via phi29‐mediated rolling circle amplification (RCA) to produce DNA nanoballs. DNBs were then loaded onto a patterned nanoarray for paired‐end sequencing (PE300/250) on the DNBSEQ‐G400 platform (BGI) to generate reads.

### 5.13 Analysis pipeline for 16S rRNA gene amplicon sequencing

Raw sequencing data were first subjected to quality control using iTools (v.0.25) [83]. Primer‐matched reads were selected with Cutadapt (v2.6) [84] for removal of primer and adapter contaminants to retrieve target region fragments, followed by elimination of ambiguous base‐containing and low‐complexity reads via readfq (v1.0). For sequence clustering, two parallel approaches were employed: the first utilized FLASH (v1.2.11) [85] to assemble overlapping paired‐end reads into consensus sequences, which were then clustered into OTUs at 97% similarity via UPARSE (within the USEARCH suite) [86]; chimeric sequences were filtered using UCHIME (v4.2.40) [87] against the Gold database (v20110519), and OTU abundance tables were generated by mapping all tags to representative sequences with USEARCH GLOBAL. The second approach applied DADA2 through QIIME (v1.80) [88], where filtered paired‐end sequences were imported and denoised to generate OTU at 100% similarity, with subsequent conversion of the feature table to a readable format via QIIME tools. Taxonomic annotation of OTU representative sequences was performed using RDP Classifier (v2.2) [89], followed by exclusion of unannotated OTU and those inconsistent with the study context. Alpha diversity indices were calculated for individual samples using mothur (v1.31.2) [90], while Beta diversity analysis was conducted with QIIME.

### 5.14 Linear discriminant analysis effect size (LEfSe) analysis

All LEfSe computations were conducted on the online analysis platform maintained by the Huttenhower Laboratory, which is hosted on the Galaxy Community Hub server [91] (https://huttenhower.sph.harvard.edu/galaxy/). Default parameters were adopted unless otherwise specified, ensuring consistency with standard protocols for microbiome differential abundance analysis as established in prior studies utilizing this tool. Specifically, the Kruskal–Wallis test (*α* = 0.05) was first applied to filter out taxonomic groups with non‐significant abundance differences across sites, followed by pairwise Wilcoxon tests to confirm site‐specific enrichment. The LDA score (Log10) threshold was set to the platform's default value of 2.0 to prioritize taxonomic groups with biologically meaningful discriminative power.

### 5.15 Meta‐transcriptomic virome analysis

Hecatomb is used for meta‐transcriptomic virome analysis [92]. Briefly, the analytical process initiated with stringent preprocessing to retain only non‐contaminant biological sequences for downstream analysis, involving the removal of non‐biological sequences, host sequence depletion, redundancy reduction via clustering, generation of sequence count tables, and calculation of sequence properties; a predefined set of host genomes was available, with the option to incorporate custom genomes as needed. Taxonomic classification was conducted through an iterative search strategy using MMSeqs. 2, where query sequences from preprocessing were first aligned against a virus‐specific protein database in a translated nucleotide‐to‐protein search, and sequences assigned viral taxonomy at this stage underwent secondary validation against a broader reference database covering bacteria, plants, vertebrates, and fungi. For population‐level analysis, assembly was performed to generate contigs via a resource‐efficient strategy aiming to maximize species representation: individual assemblies were generated for each sample using MEGAHIT, reads were mapped back to these assemblies with unmapped reads subjected to an additional assembly round, resulting contigs were merged into a non‐redundant dataset using Flye, contigs were annotated with MMSeqs, and complementary pseudo‐consensus annotation was implemented by integrating read mapping data with taxonomic assignments from the sequence table to enhance the resolution of contig origins and facilitate downstream virome analysis.

### 5.16 Sample preparation for metabolome and identification of differentially expressed metabolites

Fecal samples were first thawed slowly at 4°C, and 25 mg aliquots were accurately weighed for subsequent processing. Each aliquot was supplemented with 800 μL of pre‐cooled extraction solution (methanol:acetonitrile:water = 2:2:1, v/v/v) and 10 μL of internal standard to ensure extraction efficiency and data normalization. The mixture was then ground with two steel balls using a tissue grinder machine, subjected to ultrasonic treatment in a 4°C water bath for 10 min to enhance metabolite release, and incubated at −20°C for 1 h to promote further extraction. After incubation, the samples were centrifuged at 25,000 × *g* for 15 min at 4°C, and 600 μL of the resulting supernatant was transferred to a freeze vacuum concentrator for drying. The dried residue was re‐dissolved in 600 μL of reconstitution solution (methanol:H₂O = 1:9, v/v), followed by vortexing for 1 min, another round of ultrasonic treatment in a 4°C water bath for 10 min, and centrifugation at 25,000 × *g* for 15 min at 4°C; the final supernatant was then transferred to sample vials. Quality control (QC) samples were prepared by mixing 50 μL of supernatant from each sample to assess the repeatability and stability of the LC‐MS analysis process. Sample extracts were analyzed using a Waters UPLC I‐Class Plus system (Waters) coupled with a QTRAP 6500 Plus mass spectrometer (SCIEX).

Offline mass spectrometry data were imported into Compound Discoverer 3.3 (Thermo Fisher Scientific) and annotated using the BGI Metabolome Database (BMDB), mzCloud database, and ChemSpider online database, yielding a data matrix containing metabolite peak areas and identification results. Differential metabolites were screened using the Variable Importance in Projection (VIP) values derived from the first two principal components of the PLS‐DA model. Specially, the PLS‐DA model was constructed on log2‐transformed metabolome data between the two sample groups with Pareto scaling applied, and the normality of the processed data was verified. Additionally, univariate analyses of Fold Change (FC) and Student's *t*‐test were integrated for differential metabolite identification. The criteria for defining differential metabolites were as follows: a VIP value of ≥1 in the first two principal components of the PLS‐DA model, a Fold‐Change of ≥1.2 or ≤0.83, and a *q*‐value (false discovery rate‐adjusted *p*‐value) of < 0.05.

### 5.17 Western blot

Collected chicken kidney tissues were denatured and lysed after different hours post infection. The processed samples were fractionated through 12% SDS‐PAGE gel electrophoresis, and the resolved proteins were blotted onto polyvinylidene difluoride (PVDF) membranes. After completion of the blocking step using 5% bovine serum albumin (BSA), the PVDF membranes were incubated with anti‐IBV‐N mouse monoclonal antibody (MyBioSource; catalog number: MBS660026) in TBST buffer with 1% BSA for 1 h, with the subsequent step involving incubation with HRP‐labeled goat anti‐mouse IgG(H + L) secondary antibody (Proteintech; catalog number: RGAM001) for further reaction.

### 5.18 Histopathology and immunohistochemistry

The trachea and kidney tissues were fixed, dehydrated, transparentized, and embedded in paraffin. After fixation and decalcification, the tissues were sectioned to 5 μm. H&E staining and IHC were performed separately according to the instructions of the reagent. The section was captured using a Nikon TS2 microscope (Nikon Instech).

### 5.19 Phylogenetic investigation of communities by reconstruction of unobserved states 2 (PICRUSt2)

For functional profiling in microbial analysis, Phylogenetic Investigation of Communities by Reconstruction of Unobserved States 2 (PICRUSt2) [93] was applied; this bioinformatics platform predicts microbial gene functions on the basis of 16S rRNA gene sequencing datasets. By utilizing reference genome databases, this tool infers the potential metabolic pathways encoded by the microbiota. Functional annotations of bacterial gene functions were carried out using the KEGG database. Differential abundance analysis of bacterial taxa and predicted gene functions was implemented with STAMP, where statistical significance was defined as *p* < 0.05.

### 5.20 Enzyme‐linked immunosorbent assay (ELISA) for cytokine quantification

Serum levels of inflammatory cytokines were quantified using commercial ELISA kits, following the manufacturers' protocols. Absorbance measurements were recorded at 450 nm using a microplate reader (Bio‐Rad). Specifically, IL‐16, IFN‐α, IFN‐γ, IL‐10, IL‐21, IL‐2, IL‐6, and CCL4 were analyzed using the Millipore MILLIPLEX Chicken Cytokine/Chemokine Panel 1 ELISA kit (GCYT1‐16K‐PX12). Additionally, IL‐1β (CSB‐E11230Ch), IFN‐β (CSB‐E11237Ch), and TNF‐α (CSB‐E11231Ch) levels were determined using separate ELISA kits.

### 5.21 Mantel test for correlation analysis of microbiome, virome, and metabolome

To evaluate correlations among microbiome, virome, and metabolome datasets, a Mantel test was performed, and the relationships were visualized using a correlation heatmap. The microbiome, virome, and metabolome datasets were imported into R, where Pearson correlation coefficients were computed for environmental factors. Statistically significant correlations were extracted and stored for further analysis. The Mantel test was applied to assess inter‐dataset associations, with results categorized and visualized. A correlation heatmap was generated, highlighting statistically significant Pearson correlation values. Additionally, Mantel test results were overlaid on the heatmap using connecting lines of varying thickness and color to represent correlation strength and significance.

### 5.22 Prediction of bacterial phage host range using integrated phage host prediction (iPHoP)

iPHoP (Integrated Phage HOst Prediction) was employed to predict the host range of bacterial phages [94]. All assembled phage contigs were selected and imported into iPHoP in FASTA format. The analysis was conducted using default parameters, leveraging BLAST, CRISPR, and iPHoP‐RF methodologies for host prediction. The resulting host predictions were subsequently imported into R for downstream visualization.

### 5.23 Construction and visualization of the microbiome–virome interaction network

To construct and visualize microbiome–virome interactions, raw microbiome and virome abundance data were uploaded to http://cloudtutu.com.cn/. Pearson's correlation analysis was conducted to assess relationships between microbiomic and viromic datasets, applying a filtering criterion of |*r*| ≥ 0.5 and *p* ≤ 0.05. The resulting correlation data were exported and imported into R for network visualization. A microbiome–virome interaction network was constructed using a force‐directed layout algorithm. The network graph was plotted with annotations indicating modular structures, node classifications, edge weights, and node abundances to enhance interpretability.

### 5.24 Statistical analysis

For multi‐group comparisons, the two‐way analysis of variance (ANOVA) was employed to evaluate the main effects of group, time, and their interaction for time‐course data. For comparisons involving multiple treatment groups without time factors, the one‐way ANOVA was used. Bonferroni correction was applied for post‐hoc multiple comparisons in both cases to avoid type I error inflation. For two‐group comparisons, the Student's *t* test was used. Survival analysis was conducted using the Kaplan–Meier (K–M) method to determine the significance of survival differences between groups. All statistical analyses above were performed using GraphPad Prism 10.3.0 software.

## AI STATEMENT

The preliminary version of this manuscript underwent grammar refinement and sentence structure optimization with the assistance of Doubao (https://www.doubao.com/chat/). All content generated by the AI tool was thoroughly reviewed and revised by the authors to guarantee the accuracy of facts and eliminate any potential biases caused by AI. We hereby affirm that no AI tools were utilized in the procedures of data generation, data analysis, or the creation of visual content.

## AUTHOR CONTRIBUTIONS

**Peng Ouyang:** Methodology; software; data curation; investigation; visualization; validation; formal analysis; writing—original draft; writing—review & editing. **Yufang Liu:** Methodology; data curation; investigation; validation; formal analysis; writing—original draft. **Xuanci Wang:** Software; investigation; validation. **Yihui Huang:** Software; investigation. Yiqin Yang: Investigation. **Qixiang Kang:** Investigation. **Rui Geng:** Investigation. **Guangli Hu:** Investigation. **Aiping Lv:** Investigation. **Fangyu Hu:** Investigation. **Yongbo Xia:** Investigation. **Xin Luo:** Investigation. **Jiamin Liao:** Investigation. **Yihong He:** Investigation. **Zhanpeng Kuang:** Investigation. **Yunping Du:** Investigation. **Yun Zhang:** Investigation. **Chunyi Xue:** Investigation. **Zongxi Han:** Investigation. **Wen‐Jun Li:** Resources. **Lei Dong:** Writing—review & editing; conceptualization; visualization; validation; data curation. **Hao Zhang:** Conceptualization; writing—review & editing; visualization; resources; methodology; data curation. **Yongchang Cao:** Funding acquisition; project administration; resources; writing—review & editing; conceptualization; supervision. All authors have read the final manuscript and approved it for publication.

## CONFLICT OF INTEREST STATEMENT

The authors declare no conflicts of interest.

## ETHICS STATEMENT

All sample collection and animal experiments in this study were conducted in accordance with the guidelines of the Animal Ethics Committees of Sun Yat‐sen University and Harbin Veterinary Research Institute (HVRI) (210512‐08 and 230721‐02‐GR).

## Supporting information


**Figure S1.** Viral loads and pathological analysis following IBV infection in adult and young chickens.
**Figure S2.** Viral loads and pathological analysis following IBV infection in FMT experiment.
**Figure S3.** Analysis of gut microbiome origins in recipient chickens following FMT from adult chickens.
**Figure S4.** Microbiome composition analysis in adult chickens, young chickens, and FMT‐treated young chickens.
**Figure S5.** LEfSe analysis between different groups.
**Figure S6.** Functional prediction of gut microbiota in adult and young chickens.
**Figure S7.** Virome composition analysis in adult chickens, young chickens, and FMT‐treated young chickens.
**Figure S8.** Analysis of assembled viral contigs in viromic data.
**Figure S9.** Prediction of host targets for phage contigs assembled from the virome.
**Figure S10.** Microbiome and virome interaction networks in the gut.
**Figure S11.** Metabolomic analysis of gut samples from adult chickens, young chickens, and FMT‐treated young chickens.
**Figure S12.** 16S rRNA gene sequencing analysis of gut microbiome in young chickens before and after IBV infection.
**Figure S13.** Relative abundance of upregulated bacteria after IBV infection in healthy adult, young or young‐FMT chickens.
**Figure S14.** Evaluation of the safety of candidate transplanted bacteria strains in chickens and hematoxylin‐eosin (H&E) staining tissue section analysis following oral gavage of *A. muciniphila*.
**Figure S15.** Metabolomic analysis of the gut in young chickens administrated of *A. muciniphila* after IBV infection.
**Figure S16.** Relative abundance of GABA in different treatment groups.
**Figure S17.** Concentration of IFNs in serum and pathological analysis of trachea and kidneys following oral gavage of with *A. muciniphila* and IBV infection in young chickens.

**Table S1.** Genotypes of IBV isolates collected by our research team from 2019 to 2024.
**Table S2.** Taxonomic classification of 16S rRNA gene amplicons from adult, young, and young‐FMT samples.
**Table S3.** Comparison of predicted microbiota functions between adult and young chicken groups.
**Table S4.** Species‐level taxonomic classification of virome data from adult, young, and young‐FMT samples.
**Table S5.** Predicted host ranges of assembled phage contigs in virome data.
**Table S6.** Interaction network between microbiota and viruses in adult and young chicken groups.
**Table S7.** Expression levels and functional annotations of identified metabolites in adult, young, and young‐FMT samples.
**Table S8.** Differentially expressed metabolites between adult and young chicken groups.
**Table S9.** KEGG pathway analysis of differentially expressed metabolites between adult and young chicken groups.
**Table S10.** Taxonomic classification of 16S rRNA gene amplicons from young chickens before infection and at 3, 5, and 7 dpi.
**Table S11.** Differentially expressed metabolites in *A. muciniphila*‐treated and IBV‐infected chickens compared to the IBV‐infected group.

## Data Availability

The raw sequencing data generated in this study, including 16S rRNA gene sequencing of 100 samples and metatranscriptomic sequencing of 60 samples, have been deposited in the National Center for Biotechnology Information (NCBI) database under the accession numbers PRJNA1219767 (https://www.ncbi.nlm.nih.gov/bioproject/PRJNA1219767) and PRJNA1222581 (https://www.ncbi.nlm.nih.gov/bioproject/PRJNA1222581). The untargeted metabolomics data of 100 samples have been deposited in the MetaboLights database at the European Molecular Biology Laboratory (EMBL)–European Bioinformatics Institute (EBI) under the accession number MTBLS12310 (https://www.ebi.ac.uk/metabolights/reviewer694c2e26-ed7a-464f-a12d-c0a70f0ded83). The data and scripts for analysis can be found at https://github.com/pengouy/IMO-2026. Supplementary materials (figures, tables, graphical abstract, slides, videos, Chinese translated version, and update materials) may be found in the online DOI or iMetaOmics http://www.imeta.science/imetaomics/.
