# Supplementary material for: Akkermansia muciniphila enhances chicken resistance to infectious bronchitis virus by boosting GABA synthesis and suppressing the NF‐κB inflammatory pathway
Source: IMetaOmics. 2026 Feb 23;3(1):e70086. doi: 10.1002/imo2.70086 (PMC13674465; doi:10.1002/imo2.70086)
Supplement: Supplementary file 1 [file IMO2-3-e70086-s001.docx]

Supporting information to

***Akkermansia muciniphila* enhances chicken resistance to infectious bronchitis virus by boosting GABA synthesis and suppressing the NF-κB inflammatory pathway**

**Running title**: *Akkermansia muciniphila* enhances chicken resistance to infectious bronchitis virus

Ouyang Peng^1#^, Yufang Liu^1#^, Xuanci Wang^1^, Yihui Huang^1^, Yiqin Yang^1^, Qixiang Kang^1^, Rui Geng^1^, Guangli Hu^1^, Aiping Lv^1^, Fangyu Hu^1^, Yongbo Xia^1^, Xin Luo^1^, Jiamin Liao^1^, Yihong He^1^, Zhanpeng Kuang^1^, Yunping Du^2^, Yun Zhang^3^, Chunyi Xue^1^, Zongxi Han^4^, Wen-jun Li^1^, Lei Dong^1,*^, Hao Zhang^1,*^, Yongchang Cao^1,*^

^1^ State Key Laboratory of Biocontrol, School of Life Sciences, Sun Yat-sen University, Guangzhou 510275, China

^2^ Guangdong Enterprise Key Laboratory for Animal Health and Environmental Control, Wen’s Foodstuff Group Co. Ltd, Yunfu 527300, China

^3^ Hainan University and Massey University International College, Haikou 570228, China

^4^ Division of Avian Infectious Diseases, the State Key Laboratory of Animal Disease Prevention and Control, Harbin Veterinary Research Institute, the Chinese Academy of Agricultural Sciences, Harbin 150069, China

^#^These authors contributed equally: Ouyang Peng and Yufang Liu;

^*^ Correspondence:

[caoych@mail.sysu.edu.cn](mailto:caoych@mail.sysu.edu.cn) (Yongchang Cao)

[zhanghao5@mail.sysu.edu.cn](mailto:zhanghao5@mail.sysu.edu.cn) (Hao Zhang)

[donglei6@mail.sysu.edu.cn (Lei Dong)](mailto:donglei6@mail.sysu.edu.cn)

**Figure S1** **Viral loads and pathological analysis following IBV infection in adult and young chickens.** (A–B) Viral loads in the trachea (A) and ileum (B) at days 3, 5, and 7 post-IBV infection (*n* = 5). (C) Necropsy images of the trachea and kidneys at 5 dpi. (D) Hematoxylin-eosin (H&E) staining tissue section analysis of the trachea and kidneys at 5 dpi (Scale bar, 100 μm). (E) Immunohistochemistry analysis of the trachea and kidneys at 5 dpi (Scale bar, 100 μm). Bar plots in (A)–(B) represent the mean ± SEM, with error bars indicating the SEM (ns indicates not significant), the Two-way ANOVA was used for comparison.

**Figure S2** **Viral loads and pathological analysis following IBV infection in FMT experiment.** (A) Viral loads in ileum at days 3, 5, and 7 post-IBV infection (*n* = 5). (B) Immunohistochemistry analysis of the trachea and kidneys at 5 dpi (Scale bar, 100 μm). (C) Necropsy images of the trachea and kidneys at 5 dpi. (D) Hematoxylin-eosin (H&E) staining tissue section analysis of the trachea and kidneys at 5 dpi (Scale bar, 100 μm). Bar plots in (A) represent the mean ± SEM, with error bars indicating the SEM (ns indicates not significant). the Two-way ANOVA was used for comparison.

**Figure S3** **Analysis of gut microbiome origins in recipient chickens following FMT from adult chickens.**

**Figure S4** **Microbiome composition analysis in adult chickens, young chickens, and FMT-treated young chickens.** (A–B) Comparison of alpha diversity indices: Shannon (A) and Simpson (B) among the groups (*n* = 20). (C–D) Gut microbiome composition at the phylum (C) and species (D) levels across different groups of chickens. (E–J) Gut microbiome composition at the phylum (E), class (F), order (G), family (H), genus (I), and species (J) levels across all samples. Box and point graphs in (B)–(E) indicate the distribution of the data, the top of the box indicates upper quartile, the bottom of the box indicates the lower quartile, the line in the box indicates median, the top line beyond the box indicates maximum, the bottom line beyond the box indicates minimum (* indicate *p* < 0.05; ns indicates not significant), the One-way ANOVA was used for comparison.

**Figure S5 LEfSe analysis between different groups.** (A–B) LEfSe analysis between young-FMT group and young group. Green and blue circles denote distinct groups, with each circle representing a microbial taxa that plays a key role in the group indicated by its color. The diameter of the circles corresponds to the relative abundance of the respective taxa. Yellow circles represent microbial groups that do not exert significant effects across different groups. From the inner to the outer ring, the circles sequentially represent species classified at the phylum, class, order, family, and genus levels. (C–D) LEfSe analysis between adult group and young-FMT group. Red and green circles denote distinct groups, with each circle representing a microbial taxa that plays a key role in the group indicated by its color. The diameter of the circles corresponds to the relative abundance of the respective taxa. Yellow circles represent microbial groups that do not exert significant effects across different groups. From the inner to the outer ring, the circles sequentially represent species classified at the phylum, class, order, family, and genus levels.

**Figure S6 Functional prediction of gut microbiota in adult and young chickens.**

**Figure S7** **Virome composition analysis in adult chickens, young chickens, and FMT-treated young chickens.** (A–B) Heatmaps showing the relative abundance of viral families at the order (A) and genus (B) levels in the gut virome across different groups.

**Figure S8** **Analysis of assembled viral contigs in viromic data.** (A) Distribution of contig lengths and similarities for different viral families assembled from the viromic data. (B) Phylogenetic tree of assembled *Picornaviridae* genomes based on whole-genome sequences. (C) Phylogenetic tree of assembled *Coronaviridae* genomes based on whole-genome sequences. (D) Phylogenetic tree of assembled *Astroviridae* genomes based on whole-genome sequences.

**Figure S9** **Prediction of host targets for phage contigs assembled from the virome.** (A) Sankey plot indicate the belonging family and target host genus of each phage contig. (B) Line plot shows the total confidence score of prediction for each contig. (C) Bar plot indicate the max score among three prediction method, BLAST, CRISPR, and iPhoP-RF.

**Figure S10** **Microbiome and virome interaction networks in the gut.** (A–B) Interaction networks between microbiota and viruses in the gut of adult chickens (A) and young chickens (B).

**Figure S11** **Metabolomic analysis of gut samples from adult chickens, young chickens, and FMT-treated young chickens.** (A) Classification of detected metabolites in chicken gut feces. (B) KEGG functions of detected metabolites. (C) Venn diagram showing overlapping enriched pathways between differential metabolites and predicted microbiome pathways among adult and young groups. (D) Correlation analysis of microbiome, virome, and metabolome data using Mantel test. Heatmap of metabolites relative expression level among groups.

**Figure S12** **16S rRNA gene sequencing analysis of gut microbiome in young chickens before and after IBV infection.** (A–D) Alpha diversity analysis of the gut microbiome in young chickens before IBV infection and at days 3, 5, and 7 post-infection, using the Chao1 (A), Ace (B), Shannon (C), and Sobs (D) (*n* = 10). (E) Heatmap of the relative abundance of gut microbiome at the phylum level in young chickens before IBV infection and at days 3, 5, and 7 post-infection. Box and point graphs in (A)–(D) indicate the distribution of the data, the top of the box indicates upper quartile, the bottom of the box indicates the lower quartile, the line in the box indicates median, the top line beyond the box indicates maximum, the bottom line beyond the box indicates minimum (**, ***, or **** indicate *p* < 0.01, *p* < 0.001, and *p* < 0.0001, respectively; ns indicates not significant), the Student’s t test was used for comparison.

**Figure S13** **Relative abundance of upregulated bacteria after IBV infection in healthy adult, young or young-FMT chickens.** (A–C) Relative abundance of the upregulated bacteria *Cloacibacillus porcorum* (A), *Neglecta timonensis* (B), and *Akkermansia muciniphila* (C) following IBV infection in young chickens, as related to the 16S sequencing data from Figure 2 (*n* = 20). Box and point graphs in (A)–(C) indicate the distribution of the data.

**Figure S14** **Evaluation of the safety of candidate transplanted bacteria strains in chickens and hematoxylin-eosin (H&E) staining tissue section analysis following oral gavage of *A. muciniphila. (***A) Schematic of the experimental design: young chickens were orally administrated with *C. porcorum*, *N. timonensis*, or *A. muciniphila*. (B) Survival curves of young chickens in different groups (*n* = 10). **(**C) H&E analysis of trachea and kidneys at 5 dpi following IBV infection in young chickens following oral gavage of *A. muciniphila* (Scale bar, 100 μm).

**Figure S15** **Metabolomic analysis of the gut in young chickens administrated of *A. muciniphila* after IBV infection.** (A) Heatmap of differential metabolites between the IBV-infected group administrated of *A. muciniphila* and the IBV-infected group treated with antibiotics. (B) Functional enrichment analysis of differential metabolites between the IBV-infected group administrated of *A. muciniphila* and the IBV-infected group treated with antibiotics.

**Figure S16** **Relative abundance of GABA in different treatment groups.** (A) Relative abundance of GABA in the IBV-infected group treated with antibiotics or the IBV-infected group administrated of *A. muciniphila* (*n* = 10). (B) Relative abundance of GABA in healthy adult or young chickens (*n* = 20). Point graphs in (A)–(B) indicate the distribution of the data, the black lines indicate mean (***, or **** indicate *p* < 0.001, and *p* < 0.0001, respectively), the Student’s *t* test was used for comparison.

**Figure S17** **Concentration of IFNs in serum and pathological analysis of trachea and kidneys following oral gavage of with *A. muciniphila* and IBV infection in young chickens.** (A–B) Expression levels of IFN-α (A) and IFN-β (B) in serum samples collected at day 5 post-IBV infection using ELISA kits (*n* = 5). (C) Hematoxylin-eosin (H&E) staining tissue section analysis of the trachea and kidneys at 5 dpi (Scale bar, 100 μm). (D) Immunohistochemistry (IHC) analysis of the trachea and kidneys at 5 dpi (Scale bar, 100 μm). Bar graphs in (A)–(B) represent the mean ± SEM, with error bars indicating the SEM (**, or *** indicate *p* < 0.01 and *p* < 0.001, respectively; ns indicates not significant), the One-way ANOVA was used for comparison.
